# Peli1 impairs microglial Aβ phagocytosis through promoting C/EBPβ degradation

**DOI:** 10.1371/journal.pbio.3000837

**Published:** 2020-10-05

**Authors:** Jing Xu, Tao Yu, Enrica Caterina Pietronigro, Jia Yuan, Jessica Arioli, Yifei Pei, Xuan Luo, Jialin Ye, Gabriela Constantin, Chaoming Mao, Yichuan Xiao

**Affiliations:** 1 CAS Key Laboratory of Tissue Microenvironment and Tumor, Shanghai Institute of Nutrition and Health, University of Chinese Academy of Sciences, Chinese Academy of Sciences, Shanghai, China; 2 Section of General Pathology, Department of Medicine, University of Verona, Verona, Italy; 3 Department of Nuclear Medicine, The Affiliated Hospital of Jiangsu University, Zhenjiang, China; UCSD, UNITED STATES

## Abstract

Amyloid-β (Aβ) accumulation in the brain is a hallmark of Alzheimer’s disease (AD) pathology. However, the molecular mechanism controlling microglial Aβ phagocytosis is poorly understood. Here we found that the E3 ubiquitin ligase Pellino 1 (Peli1) is induced in the microglia of AD-like five familial AD (5×FAD) mice, whose phagocytic efficiency for Aβ was then impaired, and therefore *Peli1* depletion suppressed the Aβ deposition in the brains of 5×FAD mice. Mechanistic characterizations indicated that Peli1 directly targeted CCAAT/enhancer-binding protein (C/EBP)β, a major transcription factor responsible for the transcription of scavenger receptor CD36. Peli1 functioned as a direct E3 ubiquitin ligase of C/EBPβ and mediated its ubiquitination-induced degradation. Consequently, loss of Peli1 increased the protein levels of C/EBPβ and the expression of CD36 and thus, promoted the phagocytic ability in microglial cells. Together, our findings established Peli1 as a critical regulator of microglial phagocytosis and highlighted the therapeutic potential by targeting Peli1 for the treatment of microglia-mediated neurological diseases.

## Abstract 2

This study identifies Peli1, an E3 ubiqitin ligase enriched in microglia, as a restraining factor that curtails microglial phagocytosis of the amyloid Aβ. Correspondingly, deletion of Peli1 enhances Aβ phagocytosis and clearance in Alzheimer’s disease, implicating Peli1 as a therapeutic target with significant potential for the treatment of microglia-mediated neurological disease.

## Introduction

Alzheimer’s disease (AD) is a rising threat to public health worldwide [1] and is a progressive neurodegenerative disorder with the hallmark of β-amyloid (Aβ) accumulation in the brains [2,3]. Although the etiology of AD is largely unknown, the studies by analyzing the inherited AD families have suggested that multiple mutations in Aβ processing cascade are genetically associated with the disease pathogenesis [4–8]. However, human clinical trials by targeting Aβ production and deposition had failed because of the lack of efficacy in the improvement of cognitive outcomes in AD patients [9–11], which implies that the relationship between AD and Aβ plaques is still controversial and arouses the doubt on the validity about amyloid cascade hypothesis. Nevertheless, there are still numerous ongoing potential anti-Aβ antibodies therapies tested in the clinical treatment of AD, such as Gantenerumab and BAN2401. In addition, recent works also suggested that Aβ may impact the Tau hyperphosphorylation and cognitive deficit in AD-like mice, thereby affecting disease symptoms in AD patients as well [12,13]. Moreover, the anti-Aβ antibody Aducanumab has been submitted to FDA for approval, which provided a compelling support for the amyloid cascade hypothesis.

Microglia are the resident macrophages in the brain and can cluster around the amyloid plaques during AD pathogenesis. Previous studies demonstrated that microglial phagocytosis ability was associated with Aβ deposition in mouse AD models [14–19]. In contrast, microglial dysfunction is associated with aging in mice and human brains and is relevant with higher levels of Aβ load in mouse AD models [20–23]. There are also other studies demonstrated that removal of microglia by colony-stimulating factor 1 receptor (CSF1R) inhibitor prevents neuronal loss and improves cognition without affecting Aβ pathology in AD-like mice [24, 25]. These studies suggest that the pathological mechanism of AD and the function of microglia-mediated Aβ clearance are still a controversial and debated topic in the fields of neurodegenerative disorders. However, little is known about the intrinsic molecular mechanism through which Aβ is engulfed by microglial phagocytosis. We and others have previously revealed that the E3 ubiquitin ligase Pellino 1 (Peli1) is expressed in many kinds of neural cells in murine brain, with the highest expression level in the microglia [26], implying a nonredundant function of Peli1 in central nervous system (CNS) to modulate microglia-related neurological diseases. Indeed, we have identified Peli1 as a crucial modulator of microglia-mediated autoimmune neuroinflammation and viral encephalitis [26–28].

Here, we identified Peli1 as an essential negative regulator of microglial phagocytosis through inhibiting the expression of scavenger receptor CD36. Mechanistic studies revealed that Peli1 functioned as the direct E3 ubiquitin ligase of C/EBPβ, a major transcription factor that responsible for CD36 expression, and mediated its degradation. More interestingly, microglial Peli1 is induced in AD brains and thus, further impaired microglia-mediated Aβ phagocytosis during disease pathogenesis.

## Results

### *Peli1* deficiency promoted microglial phagocytic ability

**Fig 1:**
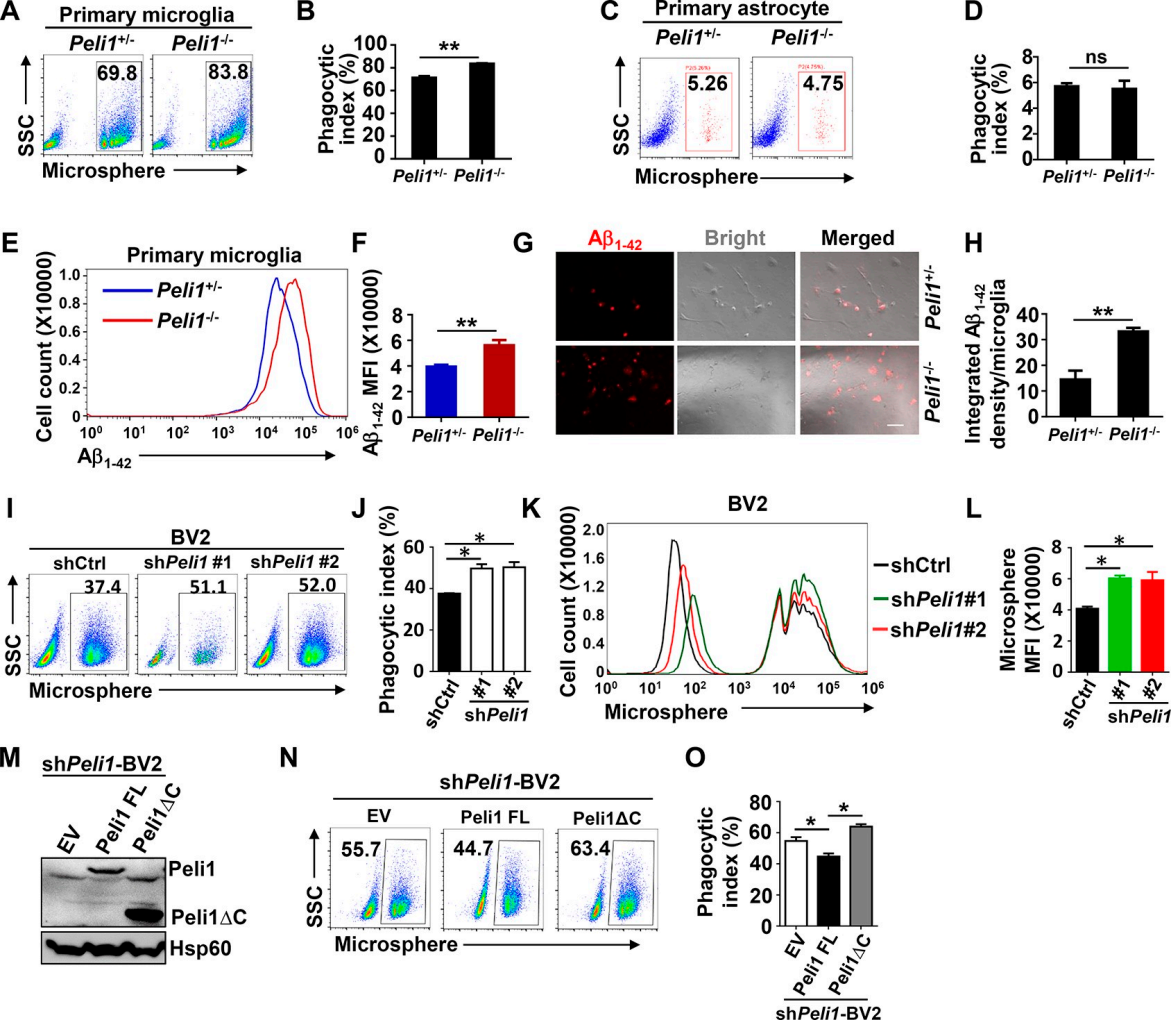
Peli1 curtails phagocytic efficiency of microglia. (**A-D**) Flow cytometry of the phagocytic ability for fluorescent microspheres in *Peli1*^+/−^ and *Peli1*^−/−^ primary microglia or astrocyte. The data are presented as representative scatter plots showing the frequencies of the cells containing fluorescent microspheres (**A, C**) and summary bar graphs (**B, D**). (**E, F**) Flow cytometry of the phagocytic ability for Rhodamine B–dyed Aβ_1–42_ peptide in *Peli1*^+/−^ and *Peli1*^−/−^ primary microglia. The data are presented as representative histogram showing the frequency of the cells containing fluorescent Aβ_1–42_ (**E**) and summary bar graph (**F**). (**G, H**) Microscopic analysis of the phagocytosis of Aβ_1–42_ peptide for 24 hours in *Peli1*^+/−^ and *Peli1*^−/−^ primary microglia. The data are presented as representative images (**G**) and summary bar graph (**H**). Scale bar: 100 μm. (**I-L**) Flow cytometry of the phagocytic ability for fluorescent microspheres in control and *Peli1*-knockdown BV2 cells. The data are presented as representative scatter plots (**I**), histogram showing the MFI (**K**) and summary bar graphs (**J, L**). (**M**) Immunoblot of Peli1 and Hsp60 (loading control) in *Peli1-*knockdown BV2 cells that reconstituted with FL Peli1 or Peli1ΔC, showing the reconstitution efficiency of Peli1. (**N, O**) Flow cytometric analysis of the phagocytic ability for fluorescent microspheres in *Peli1-*knockdown BV2 cells that reconstituted with Peli1 FL or Peli1ΔC. The data are presented as representative plots (**N**) and summary bar graph (**O**). Data with error bars represent mean ± SEM. Each panel is representative of at least 3 independent experiments. Numerical values for (B, D, F, H, J, L, O) are available in S1 Data. *P < 0.05, **P < 0.01, ***P < 0.001 as determined by unpaired Student *t* test. Aβ, amyloid-β; FL, full-length; MFI, mean fluorescent intensity; Peli1ΔC, C-terminal deleted Peli1.

We had previously reported that Peli1 could regulate microglia-mediated CNS inflammation in a multiple sclerosis mouse model [26]. However, because of the high expression of Peli1 in microglia compared with other types of cells in CNS, we speculated that Peli1 could also act as critical regulators in other physiological and pathological process in microglia. To investigate the potential role of Peli1 in modulating microglial phagocytosis, we isolated and cultured the primary microglia from the heterozygous (*Peli1*^+/−^) and *Peli1*-deficent (*Peli1*^−/−^) newborn mice from the same parents (**S1A Fig**). After challenging the primary microglia with fluorescence-labeled microspheres, we observed a robust phagocytic activity of *Peli1-*intact cells as determined by flow cytometry (**Fig 1A and 1B**). Interestingly, loss of Peli1 enhanced microglial phagocytosis of microspheres, as reflected by increased frequencies of fluorescent cells in *Peli1*-deficient microglia (**Fig 1A and 1B**). In contrast, *Peli1* deficiency does not affect the phagocytic ability of primary cultured astrocytes (**Fig 1C and 1D**). We further validated the protein levels of Peli1 in microglia and astrocytes with immunoblot, and the data showed that Peli1 was highly expressed in microglia when compared with that in astrocytes (**S1B Fig**), which supported the previous study [26] and suggested a specific and nonredundant function of Peli1 in microglia to modulate phagocytosis. Subsequent flow cytometry analysis revealed that the phagocytic microspheres-induced mean fluorescent intensity (MFI) in *Peli1*-deficient microglia was enhanced as compared with *Peli1*-competent cells (**S1C and S1D Fig**).

Although Peli1 ablation didn’t affect the ability of microglia to phagocytize 1–3 microspheres, *Peli1*-deficient microglia were more competent to phagocytize more than 4 microspheres (**S1E Fig**), suggesting that Peli1 negatively regulates microglial phagocytic efficiency. Furthermore, to confirm the function of Peli1 in regulating microglial phagocytosis, we examined the phagocytic ability for the synthetic Aβ_1–42_ peptide by using the murine primary microglia. Consistent with the data of microspheres phagocytosis, *Peli1* deficiency also enhanced the microglial ability to phagocytize Aβ_1–42_, as reflected by increased MFI of Aβ_1-42_-containing cells and elevated Aβ_1–42_ loading per microglia (**Fig 1E–1H**). In order to validate that the Aβ_1–42_ oligomers were really internalized by phagocytosis rather than endocytosis, we performed the phagocytic assay of Aβ_1–42_ peptide with Cytochalasin D to inhibit actin polymerization. As the data show, the differences of Aβ_1–42_ phagocytosis between *Peli1*-sufficient and *Peli1*-deficient microglia were totally abolished and significantly inhibited after Cytochalasin D treatment (**S1F and S1G Fig**), implying that the Aβ_1–42_ peptide entered microglia via phagocytosis but not endocytosis. The ability of microglial phagocytosis was also essential for the clearance of apoptotic neurons. Therefore, we tested the phagocytic ability of apoptotic neural Neuron-2A (N2A) cells in *Peli1-*deficient microglia. The data demonstrated that the phagocytosis of apoptotic N2A was not affected after Peli1 was deleted in microglia (**S1H and S1I Fig**), which further supported the critical and specific role of Peli1 in microglial phagocytosis of Aβ_1–42_ peptide.

To exclude the potential possibility of proliferation-induced alteration of phagocytic ability, we examined whether Peli1 modulates microglial proliferation. The results revealed that *Peli1* deficiency in primary microglia or its knockdown in BV2 microglial cells does not affect the growth of microglial cells by Ki-67 staining (**S2A–S2C Fig**). Consistent with the results obtained from primary microglia, we found that reducing Peli1 levels in BV2 cells also promoted the phagocytosis activity, as characterized by increased frequencies of fluorescent cells and enhanced microglial MFI levels when compared with control cells (**Fig 1I–1L**). In addition, we performed a time course assay of microspheres phagocytosis in BV2 cells. The results suggested that *Peli1* knockdown indeed led to increased microspheres phagocytosis after microspheres inoculation for 10, 20, 30, and 60 minutes (**S2D and S2E Fig**). Furthermore, the percentages of microsphere phagocytosis in *Peli1*-knockdown BV2 for more than 4 particles per cell were higher, whereas the BV2 percentages for 1 particle per cell were lower than that in control at all of the later time points, implying that *Peli1*-knockdown BV2 could phagocytize more throughout the whole process of phagocytosis (**S2F Fig**). Moreover, the difference of percentages between control and *Peli1*-knockdown BV2 cells that phagocytize more than 4 particles was smaller at 60 minutes than at 10 minutes (**S2D and S2F Fig**), which revealed that the rates of phagocytosis in both cells were altered with time. Consistent with the result of microspheres phagocytosis, *Peli1* knockdown in BV2 cells also enhanced the ability to phagocytize Aβ_1–42_ after incubation with Aβ for 12 hours or 24 hours (**S2G and S2H Fig**).

To further confirm the role of Peli1 in regulating microglial phagocytosis, we reconstituted *Peli1*-knockdown BV2 cells with full-length (FL) Peli1 or C-terminal-deleted (Peli1ΔC) Peli1 (**Fig 1M**), which lacked the RING-like domain and thus lost its E3 ligase activity, and assayed the phagocytic activities. The results showed that reconstitution with only FL-Peli1, but not its functional deficient Peli1ΔC, could successfully rescue the effect of Peli1 in suppressing microglial phagocytic ability (**Fig 1N and 1O**). Collectively, these data identified an intrinsic negative role of Peli1 in regulating microglial phagocytosis.

### Enhanced phagocytosis in *Peli1*-deficient microglia was dependent on C/EBPβ

**Fig 2:**
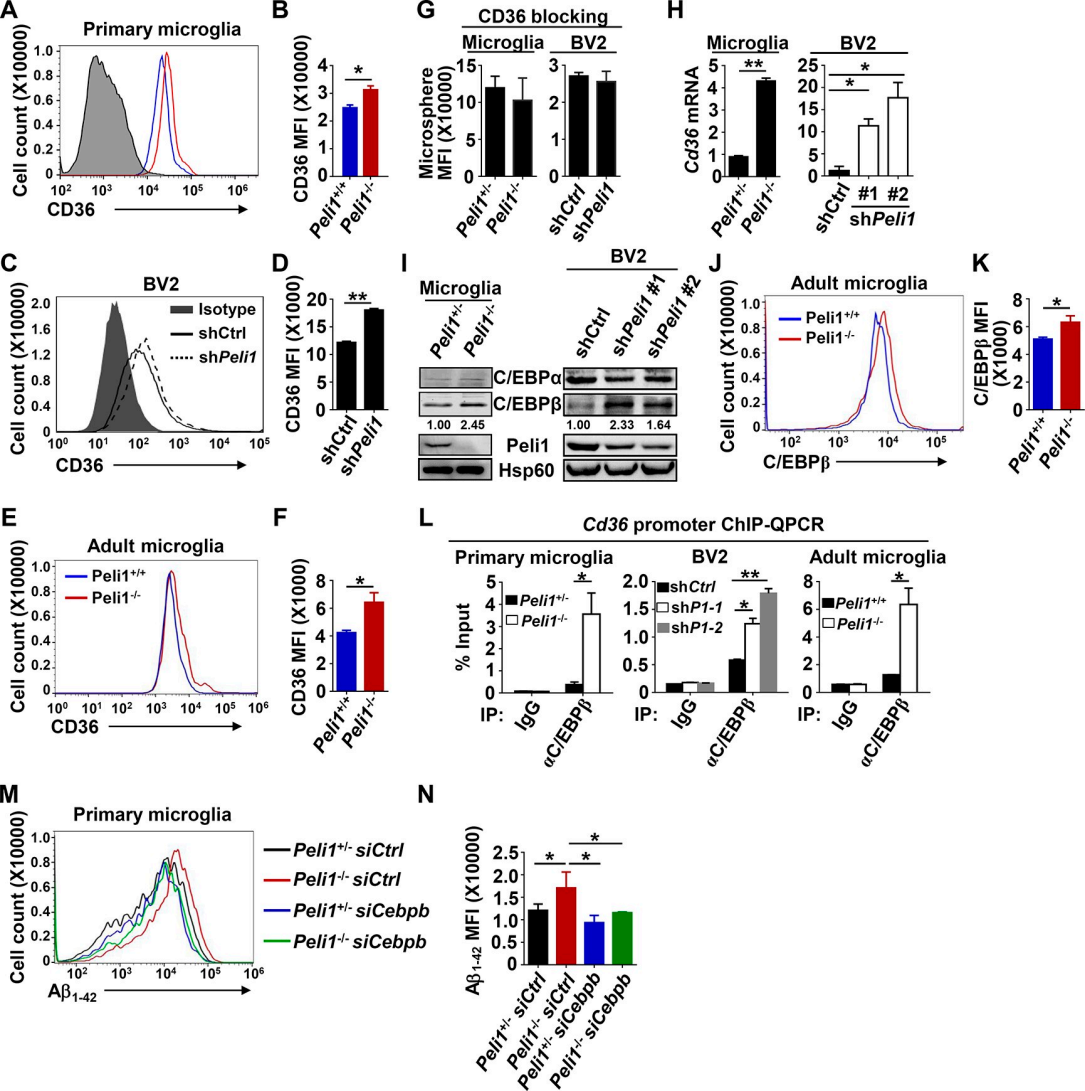
*Peli1* deficiency promotes C/EBPβ-mediated *Cd36* transcription in microglia. (**A-F**) Flow cytometry of CD36 expression on the surface of *Peli1*^+/+^ and *Peli1*^−/−^ primary microglia (**A, B**), control and *Peli1*-knockdown BV2 cells (**C, D**), and microglia isolated from age- and sex-matched adult *Peli1*^*+/+*^ and *Peli1*^−/−^ mice (**E, F**). The data are presented as representative histograms (**A, C, E**) and summary bar graphs (**B, D, F**). (**G**) Flow cytometric analysis of the phagocytic ability for fluorescent microspheres in *Peli1*^+/−^ and *Peli1*^−/−^ primary microglia and in control and *Peli1*-knockdown BV2 cells that pretreated with anti-CD36 neutralization antibody and presented as summary bar graphs. (**H**) Real-time qPCR analysis of *Cd36* mRNA expression in *Peli1*^+/−^ and *Peli1*^−/−^ primary microglia and in control and *Peli1*-knockdown BV2 cells. (**I**) Immunoblot of C/EBPα, C/EBPβ, Peli1, and Hsp60 (loading control) in *Peli1*^+/−^ and *Peli1*^−/−^ primary microglia and in control and *Peli1*-knockdown BV2 cells. (**J-K**) Flow cytometric analysis of the intracellular C/EBPβ expression in microglia isolated from *Peli1*^+/+^ and *Peli1*^*−/−*^ adult mice. The data are presented as representative histogram showing MFI of C/EBPβ staining (**J**) and summary bar graph (**K**). (**L**) ChIP-qPCR analysis of the binding activity of C/EBPβ in the promoter of *Cd36* gene after immunoprecipitation with anti-C/EBPβ antibody in *Peli1*^+/−^ and *Peli1*^−/−^ primary microglia, control and *Peli1*-knockdown BV2 cells, and microglia isolated from age- and sex-matched adult *Peli1*^+/+^ and *Peli1*^−/−^ mice. (**M, N**) Flow cytometry of the phagocytic ability for Aβ_1–42_ peptide in *Peli1*^+/−^ and *Peli1*^−/−^ primary microglia that transfected with si*Cebpb*. The data were presented as representative histogram (**M**) and summary bar graph (**N**). Data with error bars represent mean ± SEM. Each panel is representative of at least 3 independent experiments. Numerical values for (B, D, F, G, H, K, L, N) are available in S1 Data. *P < 0.05, **P < 0.01, ***P < 0.001 as determined by unpaired Student *t* test. Aβ, amyloid-β; ChIP, chromatin immunoprecipitation; IgG, immunoglobulin G; IP, immunoprecipitation; MFI, mean fluorescent intensity; qPCR, quantitative PCR.

It is known that the molecular structures of extracellular substrates are recognized by different types of scavenger receptors that are expressed on the surface of microglia, which then initiate phagocytosis for its clearance function [29–33]. To determine whether enhanced phagocytic efficiency observed in *Peli1*-deficient microglia was due to the alteration of the expression of these phagocytic receptors, we examined the surface expression of scavenger receptors class A (SRA) and CD36, the major scavenger receptors responsible for the clearance of Aβ in microglia [29,30,34]. The results revealed that *Peli1* deficiency or knockdown didn’t affect the surface expression of SRA in primary microglia or BV2 cells (**S3A–S3D Fig**). Interestingly, reducing Peli1 levels promoted CD36 expression on the surface of primary microglia, BV2 cells, and the microglia that directly isolated from the brains of adult mice (**Fig 2A–2F**). In addition, loss of Peli1 also increased the total protein levels of CD36 in these cells (**S3E–S3G Fig**), which further validated the negative role of Peli1 in regulating CD36 expression. More intriguingly, CD36 blocking with antibody totally abolished the phagocytic difference between *Peli1*-competent cells and *Peli1*-deficient primary microglia or *Peli1*-knockdown BV2 cells (**Fig 2G**), implying that elevated CD36 expression in *Peli1*-deficient or *Peli1-*knockdown cells contributed to their enhanced phagocytic ability.

To figure out how Peli1 regulates CD36 expression on the surface of microglia, we initially examined its mRNA expression in microglial cell. The results revealed that *Peli1* deficiency or knockdown enhanced the mRNA expression of *Cd36* in both primary microglia and BV2 cells (**Fig 2H**), suggesting that *Peli1-*mediated alteration of CD36 expression was directly modulated at transcriptional level. In order to exclude the potential possibility of other receptors in regulating microglial phagocytosis in *Peli1-*deficient cells, we further tested the mRNA levels of scavenger receptor B-1 (*Srb1*), macrophage receptor with collagenous structure (*Marco*), the receptor for advanced glycation end product (*Rage*), and triggering receptor expressed on myeloid cells 2 (*Trem2*), which also mediated Aβ phagocytosis as previous reported [35]. The data showed that *Peli1* deficiency did not promote the expression of SR-B1, MARCO, RAGE, or TREM2 in murine primary microglia (**S4A Fig**), implying the specific role of Peli1 in regulating *Cd36* transcription.

The mRNA expression of *Cd36* was previously reported to be tightly controlled by 2 major transcription factors, CCAAT/enhancer-binding protein (C/EBP) α and C/EBPβ, which could directly bind to the *Cd36* gene promoter and initiate its mRNA transcription [36]. Although C/EBPα was more potent than C/EBPβ to induce *Cd36* transcription, our results revealed that *Peli1* deficiency in microglia or *Peli1* knockdown in BV2 cells did not induced C/EBPα protein expression (**Fig 2I**). In contrast, *Peli1* deficiency or knockdown significantly increased the protein levels of C/EBPβ in both primary microglia and BV2 cells (**Fig 2I, S4B and S4C Fig**). In addition, we confirmed that increased C/EBPβ protein expression was also observed in *Peli1*-deficent adult microglia as compared with that in wild-type (WT) cells (**Fig 2J and 2K**). Accordingly, the chromatin immunoprecipitation (ChIP)-quantitative PCR (qPCR) analysis verified that there were indeed more C/EBPβ protein binding in the *Cd36* gene promoter of *Peli1*-deficient primary microglia, adult microglia, and *Peli1*-knockdown BV2 cells than that in control cells (**Fig 2L**). These results suggested that Peli1 modulated the transcription of scavenger receptor *Cd36* by specifically targeting C/EBPβ protein expression in microglial cells.

To confirm that increased CD36 expression and phagocytic activity in *Peli1*-deficient microglia is due to elevated expression of C/EBPβ, we reduced *Cebpb* expression in microglial cells. The results revealed that knockdown of *Cebpb* compromised the increased phagocytosis of Aβ_1–42_ and microspheres in both *Peli1*-deficient primary microglia and *Peli1*-knockdown BV2 cells (**Fig 2M and 2N, S5A–S5D Fig**). Unexpectedly, *Cebpb* knockdown failed to further inhibit the phagocytic activity in *Peli1*-competent cells (**S5C and S5D Fig**), implying the phagocytosis might be compensated by other scavenger receptors in the absence of CD36 that induced by *Cebpb* knockdown. Moreover, knockdown of *Cebpa* didn’t affect the phagocytic activity and CD36 expression in both control and *Peli1*-knockdown BV2 cells (**S5E–S5G Fig**), which confirmed the dispensable role of C/EBPα in regulating *Peli1-*mediated inhibition of microglial phagocytosis. Therefore, these data collectively demonstrated that C/EBPβ was important for the enhanced CD36-mediated phagocytosis in microglia with Peli1 absent.

### Peli1 mediated the ubiquitination and degradation of C/EBPβ

**Fig 3:**
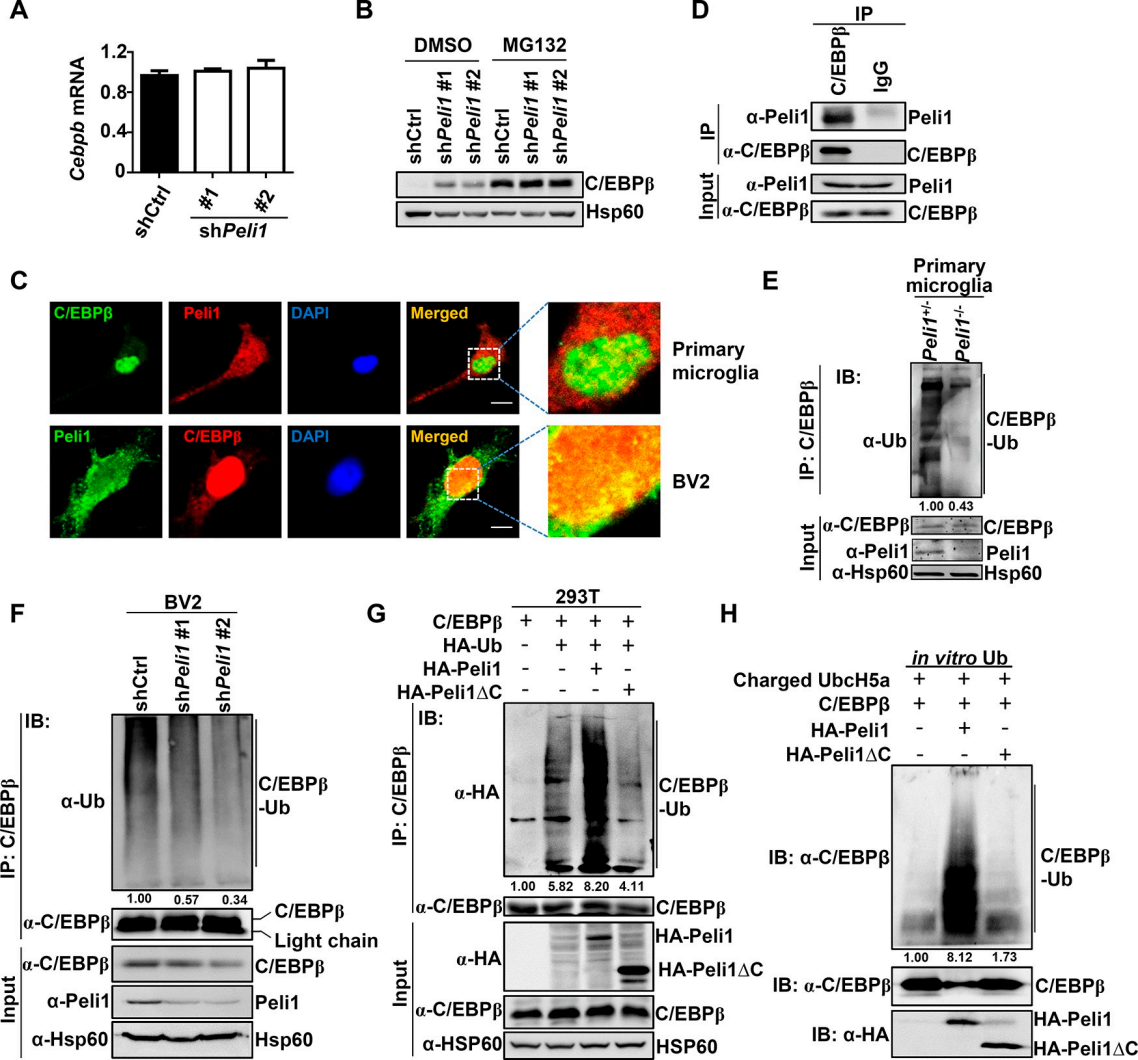
Peli1 mediates the ubiquitination and degradation of C/EBPβ. (**A**) qPCR analysis of *Cebpb* mRNA expression in control and *Peli1*-knockdown BV2 cells. (**B**) Immunoblot of C/EBPβ and Hsp60 (loading control) in control and *Peli1*-knockdown BV2 cells that pretreated with or without a proteasome inhibitor MG132 for 4 hours. (**C**) Immunofluorescent images showing the intracellular colocalization of Peli1 and C/EBPβ in primary microglia and BV2 cells. The zoomed images indicated the colocalization of Peli1 and C/EBPβ in the nucleus of the cells. Scale bars: 10 μm. (**D**) Immunoassays on lysates of BV2 cells after immunoprecipitation with control IgG or anti-C/EBPβ and immunoblot analysis of C/EBPβ associated Peli1. (**E, F**) Ubiquitination of endogenous C/EBPβ in *Peli1*^+/−^ and *Peli1*^−/−^ primary microglia (**E**) and in control and *Peli1*-knockdown BV2 cells (**F**) that were pretreated with MG132 for 4 hours, assessed by immunoblot analysis with anti-ubiquitin and anti-C/EBPβ after immunoprecipitation with anti-C/EBPβ (top), and by immunoblot analysis with input proteins and loading control (below). (**G**) Ubiquitination of C/EBPβ in 293T cells transfected with the indicated expression vectors, assessed by immunoblot analysis with anti-HA after immunoprecipitation with anti-C/EBPβ (top) or by immunoblot analysis with input proteins in lysates without immunoprecipitation (below). (**H**) In vitro ubiquitination assay of C/EBPβ ubiquitination after a mixture reaction of ubiquitin-charged E2 UbcH5a, in vitro translated C/EBPβ, and with or without HA-Peli1 or HA-Peli1ΔC proteins. Data with error bars represent mean ± SEM. Each panel is representative of at least 3 independent experiments. Numerical values for (A) are available in S1 Data. C/EBP, CCAAT/enhancer-binding protein; HA, hemagglutinin; IB, immunoblot; IgG, immunoglobulin G; IP, immunoprecipitation; Peli1ΔC, C-terminal-deleted Peli1; qPCR, quantitative PCR; UbcH5a, ubiquitin-conjugating enzyme H5a.

To dissect the molecular mechanism through which Peli1 regulated C/EBPβ expression, we firstly examined the mRNA levels of *Cebpb*. The results revealed that reducing Peli1 expression does not alter the *Cebpb* mRNA levels in BV2 cells (**Fig 3A**), implying Peli1 might modulate C/EBPβ expression directly at protein level. Indeed, the C/EBPβ protein levels are comparable between control and *Peli1*-knockdown BV2 cells in the presence of a proteasome inhibitor MG132 (**Fig 3B**), suggesting Peli1 might regulate the protein stability of C/EBPβ.

We and others have previously demonstrated that Peli1 was an E3 ubiquitin ligase mediating protein activation or stability through adding the poly-ubiquitin chains to the substrates [26,37–39]. The increased C/EBPβ protein levels in *Peli1*-knockdown microglial cells promoted us to investigate whether Peli1 directly regulated the ubiquitination of this transcription factor. The immunofluorescent images showed that Peli1 was associated with C/EBPβ in the nucleus of both murine primary microglia and BV2 cells (**Fig 3C**), which was confirmed by the coimmunoprecipitated Peli1 when C/EBPβ was pulled down in BV2 cells (**Fig 3D**). Next, we examined the effect of Peli1 for the ubiquitination status of C/EBPβ in microglial cells, the result revealed that *Peli1* deficiency or knockdown inhibited the endogenous C/EBPβ ubiquitination in both primary microglia and BV2 cells (**Fig 3E and 3F**). In contrast, Peli1 didn’t affect the ubiquitination or protein level of C/EBPβ in astrocytes, implying the specific role of Peli1 in the regulation of protein stability of microglial C/EBPβ (**S5H Fig**). In addition, overexpression of FL-Peli1, but not Peli1ΔC, markedly enhanced C/EBPβ ubiquitination in 293T cells (**Fig 3G**), which explained why Peli1ΔC failed to rescue the suppressive effect of phagocytosis for Peli1 in BV2 cells (**Fig 1N and 1O**). Moreover, in vitro ubiquitination assay indicated that FL-Peli1 but not its RING-deletion mutant mediated the ubiquitination of C/EBPβ in the presence of precharged E2 ubiquitin-conjugating enzyme H5a (UbcH5a) (**Fig 3H**). Together, these results suggested that Peli1 directly associated with C/EBPβ and mediated the poly-ubiquitination and degradation of C/EBPβ in microglial cells.

### *Peli1* deficiency promoted in vivo Aβ clearance

**Fig 4:**
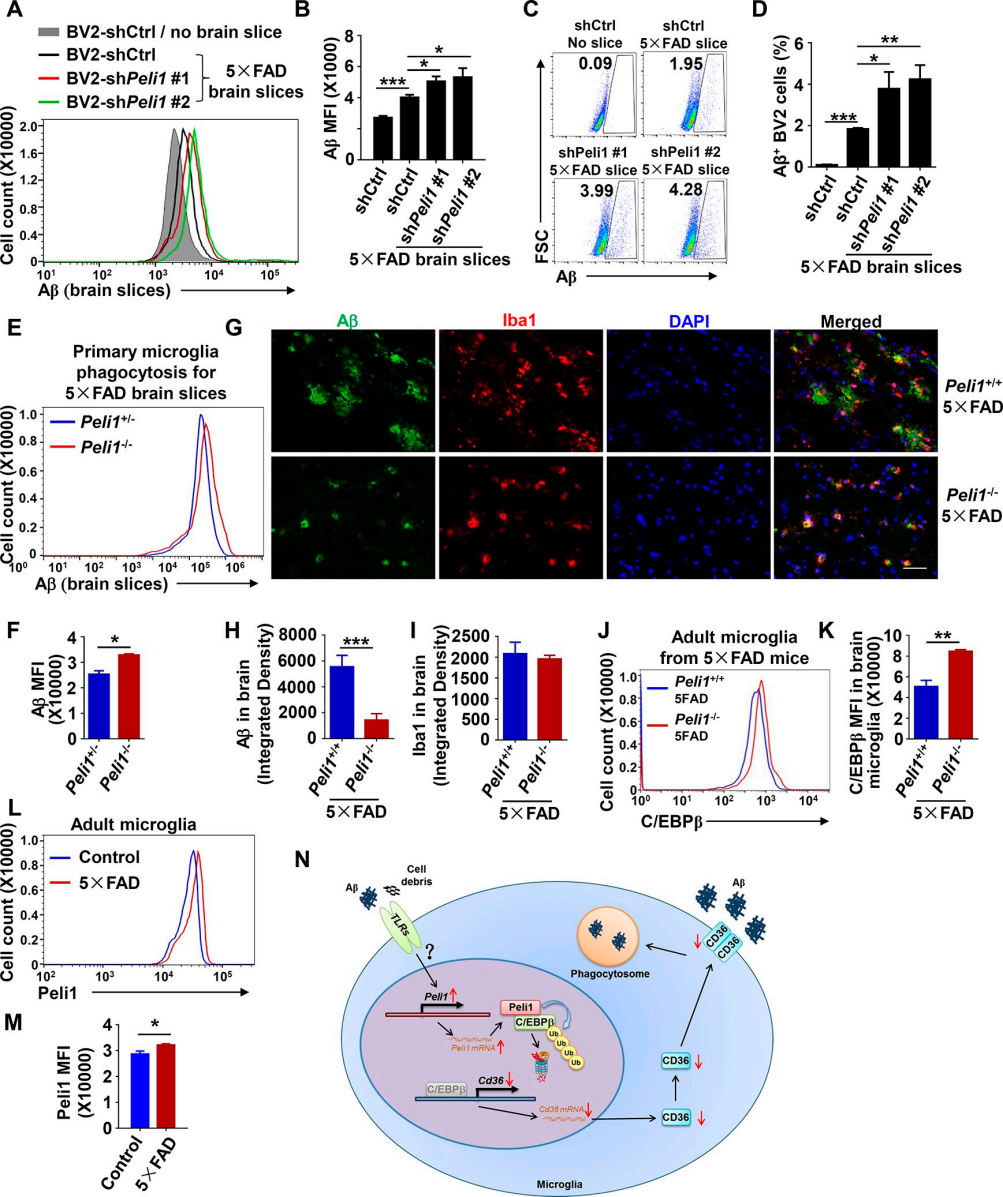
Peli1 impairs Aβ clearance in 5×FAD mice. (**A-F**) Flow cytometry of intracellular Aβ in control and *Peli1*-knockdown BV2 cells or in *Peli1*^+/−^ and *Peli1*^−/−^ primary microglia that incubated with or without the unfixed brain slices obtained from 5×FAD mice. The data are presented as representative histograms showing cellular Aβ MFI (**A, E**), scatter plots showing the frequencies of the cells that phagocytized with Aβ (**C**) and summary bar graphs (**B, D, F**). (**G-I**) Immunofluorescent images showing Aβ deposition and Iba1^+^ microglia in the brains from aged *Peli1*^+/+^ 5×FAD or *Peli1*^−/−^ 5×FAD male mice (*n =* 4 or 5 mice/group, 2–3 slices per mouse were applied for the staining). The data are presented as representative images (**G**) and summary bar graph quantifying the brain Aβ deposition (**H**) and Iba1^+^ microglia (**I**). Scale bar: 50 μm. (**J, K**) Flow cytometry of intracellular C/EBPβ expression in microglia isolated from age- and sex-matched aged *Peli1*^+/+^ 5×FAD or *Peli1*^−/−^ 5×FAD mice. The data are presented as representative histogram (**J**) and summary bar graph (**K**). (**L, M**) Flow cytometry of intracellular Peli1 in microglia isolated from 10-month-old age- and sex-matched adult naive and 5×FAD transgenic mice. The data are presented as histogram (**L**) and summary bar graphs (**M**). (**N**) Model of Peli1 in regulating microglia Aβ phagocytosis during AD pathogenesis. Data with error bars represent mean ± SEM. Each panel is representative of at least 3 independent experiments. Numerical values for (B, D, F, H, I, K, M) are available in S1 Data. *P < 0.05, **P < 0.01, ***P < 0.001 as determined by unpaired Student *t* test. 5×FAD, AD, five familial Alzheimer’s disease; Aβ, amyloid-β; MFI, mean fluorescent intensity; C/EBP, CCAAT/enhancer-binding protein.

Because CD36 is critical for the microglial phagocytosis of Aβ, we next examined whether the phagocytosis of physiological Aβ was enhanced in *Peli1*-deficient microglial cells. To this end, we performed an ex vivo Aβ phagocytosis experiment [29,30,40], in which microglial cells were added to incubate with unfixed brain slices from 10-month-old five familial AD (5×FAD) transgenic mice, an accelerated mouse model mimicking the symptoms of AD [41]. These brain slices contained numerous Aβ depositions, and thus were usually used to measure the microglial phagocytosis of physiological Aβ. As the data show, control BV2 cells efficiently phagocytized Aβ after incubation with brain slices of 5×FAD transgenic mice, as determined by intracellular flow cytometric staining of Aβ (**Fig 4A and 4B**). Interestingly, reducing Peli1 expression further promoted the phagocytic ability of Aβ from 5×FAD brain slices in both BV2 and primary microglial cells, as reflected by enhanced Aβ MFI and increased frequencies of Aβ^+^ cells (**Fig 4A–4F**).

To confirm the in vivo Peli1 function in modulating microglial phagocytosis-induced Aβ clearance, we generated WT and *Peli1*^−/−^ mice with the 5×FAD background by crossing *Peli1*^−/−^ mice with 5×FAD transgenic mice (**S6A and S6B Fig**), and then analyzed the Aβ deposition in the brains of *Peli1*^−/−^ 5×FAD mice in comparison with that of aged-matched *Peli1*^+/+^ 5×FAD mice. Interestingly, we found that *Peli1* deficiency does not affect the production of amyloid precursor protein (APP) in the brains of both 6-month-old and 10-month-old 5×FAD mice (**S6C–S6E Fig**). In 10-month-old mice, we observed a large number of Aβ were deposited in the brains of aged *Peli1*^+/+^−5×FAD mice (**Fig 4G and 4H**). Consistent with the ex vivo phagocytosis data, *Peli1* deficiency led to an obvious decrease of the Aβ stained-area along with similar staining of Iba1^+^ microglia in the aged brain slices as compared to that of WT 5×FAD mice (**Fig 4G–4I**). In addition, *Peli1* deficiency also increased C/EBPβ and CD36 proteins levels in the adult microglia that isolated from the brains with 5×FAD background (**Fig 4J and 4K, S6F and S6G Fig**). Therefore, these data confirmed the negative role of Peli1 in regulating the C/EBPβ degradation, CD36 expression, Aβ phagocytosis and Aβ clearance in 5×FAD mice.

The critical role of Peli1 in microglia to curtail Aβ phagocytosis promoted us to ask an intriguing question that whether Peli1 expression was modulated during AD pathogenesis. We then isolated the CD11b^+^ microglia from the brains of age- and sex-matched 8-month-old adult naive and 5×FAD mice and examined the Peli1 expression. Interestingly, the protein levels of Peli1 were elevated in the adult microglia of aged 5×FAD mice when compared with aged naive mice (**Fig 4L and 4M**) or in the human AD brain when compared with non-AD brain (**S6H Fig**), suggesting Peli1 was increased in the brain during AD pathogenesis. Accordingly, the expression of C/EBPβ and CD36 were decreased in the adult microglia of 5×FAD mice when compared with aged naive mice (**S6I–S6M Fig**). Together, these results suggested that the AD brain niche was a potential inducer of microglial Peli1 protein, which may, in turn, have aggravated the disease severity through regulating C/EBPβ degradation and impairing CD36 expression and microglial Aβ clearance.

## Discussion

Microglia are critical to maintain the brain homeostasis of healthy individual. Under pathological conditions, microglia functions as a damage sensor to clear cell debris and misfolded or aggregated proteins like Aβ through its abundantly expressed surface scavenger receptors [29,30]. Two scavenger receptors, SRA and CD36, are highly expressed on microglial surface and have been identified to critically regulate microglia-mediated Aβ phagocytosis and clearance [29,30,34]. In the present study, we identified Peli1, an E3 ubiquitin ligase that abundantly expressed in microglia [26], as a negative regulator of CD36 through mediating the ubiquitination and degradation of C/EBPβ. As a consequence, loss of Peli1 increased microglial CD36 expression and thus, promoted in vivo Aβ clearance of AD-like mice.

Published studies have suggested that Peli1 exhibited nonredundant function in microglia [26,27] and have established TLRs signaling as a key pathway for Peli1 induction [26,42,43]. We have previously demonstrated that microglial Peli1 is a pathogenic factor to promote neuroinflammation through mediating toll-like receptors (TLRs) signaling, which, in turn, contributed to the enhanced Peli1 expression in microglia during disease pathogenesis [26]. Other groups have also suggested that TLRs and the downstream kinase TANK-binding kinase 1 (TBK1)/inhibitor of kappa B kinase epsilon (IKKε) are critical for Peli1 induction and activation in macrophages [43,44]. It is known that Aβ could interact with TLRs and then activate the downstream signaling, and the debris from the dead neural cells in AD brains are also strong inducers of TLRs signaling [35,45,46]. Therefore, it is not surprising that microglial Peli1 is strongly induced in AD brains, and it is also reasonable to speculate that the Peli1 induction is mediated through the signaling transduced by TLRs that highly expressed on the surface of microglia during AD pathogenesis.

Microglial phagocytosis may be a double-edged sword in AD. On one hand, reduced microglial phagocytosis and suppressed clearance of apoptotic neurons and protein aggregates may result in the proinflammatory activation of microglia [47,48]. However, on the other side, inappropriate phagocytosis of neurons and synapses in neurodegeneration has been postulated, which contributes to the neuropathology of AD [49–51]. In the present study, we found that *Peli1* deficiency did not affect microglial phagocytic ability of apoptotic neuron cells (**S1H and S1I Fig**). A plausible explanation is that different scavenger receptors are involved in the phagocytosis of different substrates [31,52]. In addition, although phagocytosis of Aβ has been implicated in the spreading of toxic oligomers [53], our data showed that no spreading toxic oligomers were observed in the brain of *Peli1*-deficient mice with 5×FAD background (**Fig 4G**). These data collectively suggested that Peli1 may act specifically to regulate microglial phagocytic ability to Aβ.

In summary, our study established the E3 ligase Peli1 as a crucial regulator of microglial Aβ phagocytosis in AD. During AD pathogenesis, Peli1 expression was elevated, presumably through the activation of TLRs signaling in microglia, which increased Peli1 protein associated with the transcription factor C/EBPβ and functioned as its direct E3 ubiquitin ligase to mediate the ubiquitination and degradation of C/EBPβ. Consequently, decreased C/EBPβ impaired the transcription of the gene encoding scavenger receptor CD36 and further suppressed the phagocytic ability to Aβ of microglia (**Fig 4N**). Considering the essential pathogenic role of Peli1 in driving microglia-mediated neurodegeneration like multiple sclerosis and AD, we proposed that microglial Peli1 may be a critical novel therapeutic target that have clinical potential for the neurodegenerative diseases.

## Methods

### Ethics statement

The neuropathology studies on human brain samples were approved by the Ethical Committee from the University of Verona and Azienda Ospedaliera Universitaria Integrata di Verona (Number: protocol 20794). All the animal experimental procedures were approved by the institutional Biomedical Research Ethics Committee of Shanghai Institute of Nutrition and Health, Chinese Academy of Sciences (IACUC number: SIBS-2019-XYC-1), according to the guidelines for Animal Experiments published by the Chinese Government.

### Mice

*Peli1*^+/+^ and *Peli1*^−/−^ mice were generated by breeding *Peli1*^+/−^ mice as previously described [37]. The 5×FAD transgenic mice were purchased from Shanghai Model Organisms Center. In some experiment, *Peli1*^−/−^ mice were crossed with 5×FAD transgenic mice to generate *Peli1*^+/+^ and *Peli1*^−/−^ mice on 5×FAD background, and the age-matched male mice (10-month-old) were used to examine the Aβ deposition in the brains. All mice were maintained on a C57BL/6 background in a specific pathogen–free (SPF) facility. Mice were sacrificed by cervical dislocation or as indicated for animal experiments.

### Plasmids and reagents

The plasmids pcDNA-HA-Peli1/Peli1ΔC, PRV-Peli1/Peli1ΔC, and pLKO.1-sh*Peli1* were described as previously study [37]. The pcDNA 3.1-C/EBPβ plasmid was provided by Dr. X. Qi [54]. The pLKO.1-GFP-sh*Cebpb* plasmid was generated by cloning shRNA sequence targeting *Cebpb* to pLKO.1-GFP shRNA vector. MG132 (C2211) was purchased from Sigma, the puromycin (54041) was ordered from Merck, and FluoSpheres carboxylate-modified microsphere (1.0 μm, 2% solids, internally dyed with Nile red) (F8819) was purchased from Thermo Fisher Scientific. The anti-Peli1 (sc-271065), anti-C/EBPβ (sc-150), anti-Ubiquitin (sc-8017), and anti-Hsp60 (sc-13115) antibodies were purchased from Santa Cruz, and the anti-HA (2013819) antibody was from Roche. The PE- and Alexa Fluor 488-conjugated anti-mouse CD36 antibodies (102606 and 102608) were purchased from BioLegend, and the PE-conjugated anti-mouse CD204 (SRA) (130-102-328) antibody was from Miltenyi Biotec. The anti-β-Amyloid antibody (2454) and normal rabbit IgG (2729) were from Cell Signaling. The APC-eFluor 780-conjugated anti-mouse CD11b antibody (47-0112-82) and Alexa Fluor 488-conjugated anti-rabbit IgG secondary antibody (A11034) was from Thermo Fisher. The anti-PELI1 (12053-1-AP) and anti-GAPDH (60004-1-Ig) antibodies were from Proteintech. Antibodies were used at the concentration of 1:100 for flow cytometry, 1:1,000 for immunoblot, and 1:400 (primary antibodies) or 1:1,000 (secondary antibodies) for immunofluorescence.

### Viral infection for gene knockdown or reconstitution

BV2 cells were maintained in RPMI 1640 medium (HyClone) containing 10% fetal bovine serum (FBS, Gibco). For gene knockdown experiment, BV2 cells were infected with lentivirus containing shRNA sequence targeting mouse *Peli1* that also encoded a puromycin resistance marker or targeting mouse *Cebpb* or *Cebpa* that expressed a GFP for flow cytometric sorting. For gene reconstitution experiments, lentivirus particles containing the plasmids encoding mouse FL Peli1 orPeli1ΔC, which also expressed a GFP marker for flow cytometric sorting, were infected into the *Peli1*-knockdown BV2 cells. Eight hours after infection, virus-containing media were removed and replaced with fresh media, and the cells were selected by puromycin or sorted by flow cytometry 72 hours after infection. The puromycin selected cells or GFP^+^ cells were then applied for phagocytosis assay, immunoblotting, or qPCR analysis.

### siRNA transfection

siRNA oligonucleotides were purchased from GenePharma (Shanghai) and transfected into mouse primary microglia or BV2 cells with Lipofectamine RNAiMAX reagent (13778, Invitrogen) following the manufacturer’s protocol. In brief, siRNA was dissolved with DEPC-treated distilled water, 0.02 μM siRNA oligonucleotide and 3 μl RNAiMAX reagent were diluted with Opti-ME medium (31985062, Thermo Fisher), respectively, and incubated at room temperature for 5 minutes and then mixed (1:1) thoroughly. For the gene knockdown in primary microglia, the siRNA-RNAiMAX complex was added to the mixed glial cultures, and then the microglial cells were collected for further analysis. For the gene knockdown in BV2 cells, the siRNA-RNAiMAX complex was added to the cell carefully and cultured in cell incubator for 48 hours at 37°C, and the siRNA-transfected cells were then applied for further experiment.

### Immunoblotting and immunoprecipitation

Cells were lysed in RIPA buffer with proteinase inhibitors cocktail (B14001, Bimake), and the lysates were boiled with SDS loading buffer for 5 minutes. The immunoblotting was assessed with SDS-PAGE, followed by blotting with specific primary antibodies as well as horseradish peroxidase (HRP)-conjugated secondary antibodies. For immunoprecipitation (IP), the indicated antibody was added into the cell lysates and incubated at 4 ºC on shaker overnight after preclear, followed by pulling down the desired protein with Protein A/G magnetic beads (HY-K0202, Med Chem Express). The precipitated proteins were boiled with SDS loading buffer and assessed by immunoblotting.

### Immunofluorescence

For Peli1 and C/EBPβ colocalization assay, the murine primary microglia and BV2 cells were fixed with 4% paraformaldehyde and permeabilized with Triton X-100, followed by blocking with bovine serum albumin (Roche) at room temperature for 1 hour and incubating with specific primary antibodies at 4 ºC overnight. After washing with PBS for 3 times, the secondary antibodies labeled with different fluorescence were added and incubated for 2 hours, and the fluorescent images were assessed by confocal microscopy (ZEISS Cell Observer).

For brain Aβ deposition detection, the brain tissues were fixed with 4% paraformaldehyde overnight and dehydrated with PBS buffer containing 30% saccharose. After blocking, the brain slices were incubated with anti-Aβ, anti-APP, or anti-Iba1 primary antibodies followed by fluorescent labeled secondary antibodies, and images were assessed by microscope (ZEISS Axio Imager A2).

For microscopic analysis of the phagocytosis for Aβ_1–42_ in primary microglia and BV2 cells, the Rhodamine B–labeled oligomeric Aβ_1–42_ (2 μM) were added into the plated cells and incubated for indicated time at 37 ºC in cell incubator. The phagocytic cells were fixed with 4% paraformaldehyde at 4 ºC for 30 minutes, followed by imaging assessed with microscope (ZEISS Axio Imager A1). The integrated density of images was quantified by Image J software (NIH, https://imagej.nih.gov/ij/).

For human brain samples, paraffinated brain autopsy samples from 3 sporadic human AD patients and 3 aged-matched controls were obtained from the MRC London Brain Bank for Neurodegenerative Disease and used for immunofluorescence staining. Temporal cortex sections of male subjects were pretreated with Dako Target Retrevial Solution (pH 9, Dako, S2368) in a water bath at 95°C for 30 minutes. Brain slices were incubated in blocking buffer for 1 hour at room temperature then with anti-PELI1 antibody overnight at 4°C. After washing, appropriate fluorophore-conjugated secondary antibodies were added and then were treated with Sudan B Black 0.1% in ethanol 70% for 20 minutes. Nuclei were stained with DAPI. The sections were mounted with Dako medium. Images were acquired with Axio Imager Z2 (ZEISS, Germany).

### ChIP-qPCR assay

ChIP assay was operated under the manufacturer’s protocol (EZ-ChIP kit, 17-371*RF*, Millipore). In brief, primary microglia, adult microglia, or BV2 cells were fixed with 1% formaldehyde (F1635-500ML, Sigma-Aldrich) on the rotator for 10 minutes at room temperature and then quenched with 125 mM glycine on ice for another 10 minutes. After washing with PBS twice, cells were lysed with SDS lysis buffer, then cell lysates were sonicated with Covaris E220 for 10 minutes and precleared with Protein A agarose (16–157, Millipore). The sonicated and precleared cell lysates were immunoprecipitated with anti-C/EBPβ antibody (sc-150, Santa Cruz) or isotype IgG as control. After washing with buffers, chromatin was eluted from the Protein A/antibody/protein/DNA complex and reversed crosslinks with 5 M NaCl for 4 hours at 65 ºC. The freed DNA was digested with proteinase K and RNase A and purified with Phenol-Chloroform-Isoamyl Alcohol Mixture (Sangon Biotech). The DNA was then subjected to qPCR with SYBR Green master mix (04913914001, Roche). Sequences of ChIP-qPCR primers on mouse *Cd36* promoter were listed on **S1 Table**.

### Flow cytometry

Cell suspensions of isolated primary microglia, adult microglia, or BV2 cells were stained with the indicated antibodies or challenged with fluorescent microspheres and then subjected to flow cytometric analysis by using a Beckman Gallios flow cytometer as described before [55]. For the intracellular staining of Peli1, C/EBPβ, and Aβ, the cells were fixed and permeabilized by fixation/permeabilization buffer (00-5223-56 and 00-5123-43, Thermo Fisher) before staining the specific primary antibodies and followed by fluorescent-labeled secondary antibodies staining, and then detected by flow cytometer.

### Culture and isolation of mouse primary microglia

Primary microglia were cultured and isolated as previously described [56]. Briefly, the brains of newborn mice were dissociated with 0.25% trypsin after removing the meninges, cells were filtered using a 70-μm mesh and then cultured in complete DMEM/Ham’s F-12 medium (DF-12, HyClone). After 2 weeks of cultivation with medium changes every 3 days, the adherent astrocytes were removed through mild trypsinization for 30 minutes, and the remaining microglia were collected and were used for further experiments. The purity of the isolated microglia was determined by flow cytometry as more than 97%.

### Isolation of mouse adult microglia from brains

Adult mouse microglia were isolated as previously described [57]. In brief, 12-week-old mice were euthanized and perfused with 20 mL cold PBS. Brains were then rinsed, minced, and digested at 37°C with collagenase IV (0.5 mg/ml; Gibco) and DNase I (10 μg/ml; Roche) in RPMI 1640. Cell suspension was layered onto a Percoll (Amersham) density gradient and isolated by collection of the interface fraction between 37% and 70% Percoll according to the manufacturer’s instructions. After intensive washing and cell separation using CD11b MicroBeads (130-049-601, Miltenyi Biotec), the isolated CD11b^+^ adult microglia were then applied for further analysis by flow cytometry using specific antibodies. CD11b^+^ adult microglia could also be isolated by Adult Brain Dissociation Kit (130-107-677, Miltenyi Biotec) companied with MicroBeads separation according to the manufacturer’s procedure.

### Preparation of oligomeric Aβ_1–42_

The hexafluoroisopropanal (HFIP)-pretreated Rodamin B-labeled synthetic β-Amyloid peptide (Aβ_1–42_) was purchased from GL Biochem (Shanghai). The lyophilized Aβ_1–42_ was dissolved in dimethyl sulfoxide (D2650, Sigma) to a concentration of 2 mM and then was further diluted with Ham’s Nutrient Mixture F12 medium (51651C, Sigma) to a final concentration of 200 μM. The diluted Aβ_1–42_ was incubated at 4°C for 24 hours, then aliquoted and stored at −80°C for use.

### In vitro phagocytosis assay

Mouse primary microglia or BV2 cells were cultured in 12-well plates at a density of 1×10^5^ cells/well in RPMI 1640 with 10% FBS, and the microspheres (10 microspheres per cell; 1 μm, internally dyed with Nile red; F8819, Thermo Fisher) or Aβ_1–42_ oligomers (2 μM) were added. Cells and microspheres or Aβ_1–42_ were incubated together for indicated time points at 37°C in the cell incubator and were subsequently washed with PBS, followed by fixing with 4% paraformaldehyde for 30 minutes. The cells were then applied for flow cytometric analysis.

For apoptotic neurons phagocytosis assay, the protocol was modified as described before [58,59]. In brief, the N2A cells were digested by 0.25% trypsin and harvested by centrifugation. Collected cells were suspended with DMEM medium and incubated at 60°C for 1 hour. The N2A cells were then incubated with fixable violet dye to stain the apoptotic cells, followed by flow cytometry to make sure that the percentage of apoptotic cells was more than 95%. After washed with PBS, the stained N2A cells were added to BV2 cells in 6-well plates with DMEM medium containing 0.5% FBS at approximately 60% confluence. After incubation for 24 hours with light avoiding, the BV2 cells were harvested and stained with CD11b antibody to specifically mark the microglial BV2 cells. Percentage of phagocytic dead N2A cells in BV2 was further analyzed by flow cytometric analysis.

### Ex vivo Aβ phagocytosis assay

Ex vivo Aβ phagocytosis assays were performed as previously described [29,30]. Briefly, 10-month-old 5×FAD transgenic mice were euthanized and perfused with 20 ml cold PBS. Brains were removed from these mice and snap-frozen in cold isopentane (Sinopharm). The frozen brains sections (10 μm) were prepared and dried for 2–3 hours in the air. After washing with hybridoma serum-free medium (H-SFM; Gbico) containing 1% FBS, brain sections were cultured with 5×10^5^ BV2 cells in 1% FBS H-SFM for 18 hours at 37°C in the cell incubator. The cells were then washed for 3 times with PBS and digested with 10 mM EDTA-PBS buffer containing 2% FBS and were subsequently assessed the intracellular phagocytic Aβ by flow cytometric analysis.

### Ubiquitination assay

In vivo and in vitro ubiquitination assay were performed as previously described [60]. For C/EBPβ in vivo ubiquitination assay, the BV2 cells or 293T cells that transfected with the indicated expression vectors were treated with MG132 for 4 hours and then were lysed with lysis buffer containing protease inhibitors and N-ethylmaleimide (Sigma). The cell extracts were boiled for 5 minutes in the presence of 1% SDS to dissociate the C/EBPβ-interacting proteins and then were diluted with lysis buffer until the concentration of SDS was 0.1% before immunoprecipitation. C/EBPβ was then immunoprecipitated from the cell extracts and was assessed the status of ubiquitination with anti-ubiquitin antibody. For C/EBPβ in vitro ubiquitination assay, the HA-Peli1, HA-Peli1ΔC, and C/EBPβ were translated in vitro by using cell-free quick coupled transcription/translation system (L1170, Promega). The ubiquitination reaction was processed by mixing the translated proteins with precharged ubiquitin-conjugating enzyme E2 UbcH5a (E2-800, Boston Biochem) and incubating at 37°C for 4 hours according to the manufacturer’s protocol. The reaction was then terminated by boiling for 5 minutes in SDS loading buffer, and the samples were subjected to SDS-PAGE and immunoblotting to detect the ubiquitination of C/EBPβ mediated by Peli1.

### Real-time qPCR

Total RNA was extracted by using TRIzol reagent (15596018, Thermo Fisher Scientific), and the cDNA was reverse transcript with PrimeScript RT reagent kit (RR037A, Takara). Real-time qPCR was performed in triplicate by using SYBR Green Supermix (04913914001, Roche). The expression of individual genes was calculated and normalized to the expression of *Actb*. The gene-specific qPCR primers were listed on **S1 Table**.

### Statistical analysis

Statistical analyses were measured by GraphPad Software. Differences between groups were established using an unpaired Student *t* test for 2 conditions, a 1-way ANOVA with a Tukey’s test for multiple comparisons. *P* values of less than 0.05 were considered as statistically significant.

## Supporting information

S1 Fig Peli1 impairs phagocytosis of microspheres and Aβ_1–42_ peptide in primary microglia. (**A**) Immunoblot of Peli1 and Hsp60 (loading control) in isolated heterozygous (*Peli1*^+/−^) and *Peli1*-deficient (*Peli1*^−/−^) murine primary microglia, showing the deleting efficiency of Peli1. (**B**) Immunoblot of Peli1 and Gapdh (loading control) in isolated wild-type microglia and astrocyte, showing the expression of Peli1. (**C-E**) Flow cytometry of the phagocytic ability for fluorescent microspheres in *Peli1*^+/−^ and *Peli1*^−/−^ primary microglia. The data are presented as representative histogram showing the MFI and the relative phagocytosis efficiency of microglia (**C**) and summary bar graphs (**D, E**). (**F-G**) Flow cytometric analysis of the phagocytic ability for Aβ_1–42_ in *Peli1*^+/−^ and *Peli1*^−/−^ primary microglia treated with DMSO or actin polymerization inhibitor Cytochalasin D (5μM) throughout Aβ_1–42_ incubation. The data are presented as representative histogram showing the MFI and the relative phagocytosis efficiency of microglia (**F**), and summary bar graphs (**G**). (**H-I**) Flow cytometric analysis of the phagocytic ability for apoptotic N2A cells in *Peli1*^+/+^ and *Peli1*^−/−^ primary microglia. The data are presented as scatter plots showing the frequencies of the microglia that phagocytized with apoptotic N2A (**H**) and summary bar graph (**I**). Data with error bars represent mean ± SEM. Each panel is representative of at least 3 independent experiments. Numerical values for (D, E, G, I) are available in S1 Data. *P < 0.05, **P < 0.01, ***P < 0.001 as determined by unpaired Student *t* test. MFI, mean fluorescent intensity; N2A, Neuron-2A; ns, not significant.
(TIF)

S2 Fig Peli1 impairs phagocytosis of microspheres and Aβ_1–42_ peptide in BV2 cells. (**A**) Flow cytometric analysis of Ki67 expression in *Peli1*^+/−^ and *Peli1*^−/−^ primary microglia, the data are presented as representative histogram showing the MFI and the relative expression of Ki67 in cells. (**B**) Immunoblot of Peli1 and Hsp60 (loading control) in BV2 microglial cells with lentivirus encoding shRNA targeting *Peli1* or control, showing the deleting efficiency of Peli1. (**C**) Flow cytometric analysis of Ki67 expression in *Peli1*-sufficient and *Peli1*-knockdown BV2 cells, the data are presented as representative histogram showing the MFI and the relative expression of Ki67 in cells. (**D-F**) Flow cytometry of the phagocytic ability for microspheres incubated with control or *Peli1*-knockdown BV2 cells for indicated times. The data are presented as representative histogram showing cellular microspheres MFI (**D**), and summary bar graphs showing the MFI (**E**) and the frequencies of cells that phagocytized with microspheres (**F**). (**G, H**) Microscopic analysis of the phagocytosis of Aβ1–42 peptide at the indicated time points in control and *Peli1*-knockdown BV2 cells. The data are presented as representative images (**G**) and summary bar graph (**H**). Scale bars: 100 μm. Data with error bars represent mean ± SEM. Each panel is representative of at least 3 independent experiments. Numerical values for (A, C, E, F, H) are available in S1 Data. *P < 0.05, **P < 0.01, ***P < 0.001 as determined by unpaired Student *t* test. **P < 0.01, ***P < 0.001 as determined by unpaired Student *t* test. Aβ, amyloid-β; MFI, mean fluorescent intensity; shRNA, short hairpin RNA.
(TIF)

S3 Fig Deficiency of Peli1 increases the expression of CD36 but not SRA in microglia. (**A-D**) Flow cytometry of the SRA expression on the surface of *Peli1*^+/−^ and *Peli1*^−/−^ microglia or control and *Peli1*-knockdown BV2 cells. The data are presented as representative histogram showing SRA MFI (**A, C**) and summary bar graphs (**B, D**). (**E-G**) Immunoblot of CD36, Peli1, and Gapdh (loading control) in *Peli1*-sufficient (*Peli1*^+/+^) and *Peli1*-deficient (*Peli1*^−/−^) primary microglia, control and *Peli1-*knockdown BV2 cells, and microglia isolated from *Peli1*^+/+^ and *Peli1*^−/−^ adult mice. The data showed the increased expression of CD36 in *Peli1-*deficient cells. Data with error bars represent mean ± SEM. Each panel is representative of at least 3 independent experiments. Numerical values for (B, D) are available in S1 Data. MFI, mean fluorescent intensity; SRA, scavenger receptors class A.
(TIF)

S4 Fig Deficiency of Peli1 increases the transcription of CD36, rather than other receptors, by up-regulating C/EBPβ protein level in microglia. (**A**) Real-time qPCR analysis of *Marco*, *Srb1*, *Rage*, and *Trem2* mRNA expressions in *Peli1*^+/+^ and *Peli1*^−/−^ primary microglia. (**B-C**) Flow cytometric analysis of the intracellular C/EBPβ expression in *Peli1*^+/+^ and *Peli1*^−/−^ primary microglia. The data are presented as representative histogram showing MFI of C/EBPβ staining (**B**) and summary bar graph (**C**). Data with error bars represent mean ± SEM. Each panel is representative of at least 3 independent experiments. Numerical values for (A, C) are available in S1 Data. *P < 0.05 as determined by unpaired Student *t* test. C/EBP, CCAAT/enhancer-binding protein; *Marco*, macrophage receptor with collagenous structure; MFI, mean fluorescent intensity; qPCR, quantitative PCR; *Rage*, receptor for advanced glycation end product; *Srb1*, scavenger receptor B-1; *Trem2*, triggering receptor expressed on myeloid cells 2.
(TIF)

S5 Fig Peli1 suppresses the ability of microglial phagocytosis through regulating C/EBPβ rather than C/EBPα. (**A**) qPCR analysis of *Cebpb* mRNA expressions in *Peli1*^+/−^ and *Peli1*^−/−^ primary microglia electrotransfected with siRNA targeting *Cebpb* or control. (**B**) Immunoblot of C/EBPβ, Peli1, and Gapdh (loading control) in *Peli1*-sufficient and *Peli1*-knockdown BV2 cells with or without *Cebpb* knockdown. (**C-D**) Flow cytometry of the ability of microspheres phagocytosis in *Peli1*-sufficient and *Peli1*-knockdown BV2 cells with or without *Cebpb* knockdown. The data are presented as representative scatter plots showing the frequencies of the cells that phagocytized with microspheres (**C**) and summary bar graphs (**D**). (**E**) Immunoblot of C/EBPα and Hsp60 (loading control) in BV2 cells electrotransfected with siRNA targeting *Cebpa* or control. The data show the knockdown efficiency of C/EBPα. (**F-G**) Flow cytometric analysis of the phagocytic ability for microspheres in *Peli1*-sufficient and *Peli1*-knockdown BV2 cells with or without *Cebpa* knockdown. The data are presented as representative scatter plots showing the frequencies of the cells that phagocytized with microspheres (**F**) and summary bar graphs (**G**). (**H**) Ubiquitination of endogenous C/EBPβ in *Peli1*^+/+^ and *Peli1*^−/−^ primary astrocyte that were pretreated with MG132 for 4 hours, assessed by immunoblot analysis with anti-ubiquitin after immunoprecipitation with anti-C/EBPβ (top), and immunoblot analysis with Peli1, C/EBPβ, and loading control of whole-cell lysate in *Peli1*^+/+^ and *Peli1*^−/−^ primary astrocyte that were not pretreated with MG132 (below). Data with error bars represent mean ± SEM. Each panel is representative of at least 3 independent experiments. Numerical values for (A, D, G) are available in S1 Data. *P < 0.05, **P < 0.01 as determined by unpaired Student *t* test. C/EBP, CCAAT/enhancer-binding protein; qPCR, quantitative PCR; siRNA, small interfering RNA.
(TIF)

S6 Fig Peli1 is induced in brain microglia during AD pathogenesis. (**A-B**) The genotyping PCR analysis of *Peli1*^+/+^ and *Peli1*^−/−^ adult mice (**A**) and AD-like 5×AlD transgenic mice (**B**). (**C**) Immunoblot of APP and Gapdh (loading control) in the brain tissues of 6-month-old naive, *Peli1*^+/+^ 5×FAD and *Peli1*^−/−^ 5×FAD mice. (**D, E**) Immunofluorescent images showing APP expression in the cerebral cortex from 10-month-old *Peli1*^+/+^ 5×FAD or *Peli1*^−/−^ 5×FAD male mice (*n =* 4 or 5 mice/group). The data are presented as representative images (**D**) and summary bar graph quantifying the APP expression (**E**). Scale bar: 20 μm. (**F, G**) Flow cytometry of CD36 expression on the surface of microglia isolated from age- and sex-matched aged *Peli1*^+/+^ 5×FAD or *Peli1*^−/−^ 5×FAD male mice. The data are presented as representative histogram (**F**) and summary bar graph (**G**). (**H**) Immunofluorescent images showing PELI1 expression (green) in the brains of human AD patients and aged-matched non-AD controls. Scale bar: 30 μm. (**I-J**) Flow cytometry of the intracellular C/EBPβ expression in microglia isolated from 10-month-old age- and sex-matched adult naive and 5×FAD transgenic mice. The data are presented as representative histograms showing the MFI of the C/EBPβ staining (**I**) and summary bar graphs (**J**) quantifying the C/EBPβ expression. (**K**) qPCR analysis of *Cd36* mRNA expression in microglia isolated from 10-month-old age- and sex-matched adult naive and 5×FAD transgenic mice. (**L-M**) Flow cytometry of the surface CD36 expression in microglia isolated from 10-month-old age- and sex-matched adult naive and 5×FAD transgenic mice. The data are presented as representative histograms (**L**) and summary bar graphs (**M**). Data with error bars represent mean ± SEM. Each panel is representative of at least 3 independent experiments. Numerical values for (E, G, J, K, M) are available in S1 Data. *P < 0.05, **P < 0.01, ***P < 0.001 as determined by unpaired Student *t* test. AD, Alzheimer’s disease; APP, amyloid-beta precursor protein; C/EBP, CCAAT/enhancer-binding protein; MFI, mean fluorescent intensity; ns, not significant; qPCR, quantitative PCR; 5×FAD, five familial AD.
(TIF)

S1 Table Primers used for real-time qPCR or ChIP-qPCR. ChIP, chromatin immunoprecipitation; qPCR, quantitative PCR.
(DOCX)

S1 Data In separate sheets, the excel spreadsheet contains the numerical values for Figs 1B, 1D, 1F, 1H, 1J, 1L, 1O, 2B, 2D, 2F, 2G, 2H, 2I, 2K, 2L, 2N, 3A, 3E, 3F, 3G, 3H, 4B, 4D, 4F, 4H, 4I, 4K and 4M; S1D, S1E, S1G, S1I, S2A, S2C, S2E, S2F, S2H, S3B, S3D, S3E, S3F, S3G, S4A, S4C, S5A, S5D, S5G, S6E, S6G, S6J, S6K and S6M Figs. (XLSX)

S1 Raw images All the original images for Figs 1M, 2I, 3B, 3D, 3E, 3F, 3G and 3H; S1A, S1B, S2B, S3E, S3F, S3G, S5B, S5E, S5H, S6A, S6B and S6C Figs. (PDF)

## Abbreviations

5×FAD: five familial AD
AD: Alzheimer’s disease
Aβ: amyloid-β
C/EBP: CCAAT/enhancer-binding protein
ChIP: chromatin immunoprecipitation
CNS: central nervous system
CSF1R: colony-stimulating factor 1 receptor
FL: full-length
IKKε: inhibitor of kappa B kinase epsilon
Marco: macrophage receptor with collagenous structure
MFI: mean fluorescent intensity
N2A: Neuron-2A
Peli1: Pellino 1
Peli1ΔC: C-terminal-deleted Peli1
qPCR: quantitative PCR
Rage: receptor for advanced glycation end product
SRA: scavenger receptors class A
Srb1: scavenger receptor B-1
TLR: toll-like receptor
TBK1: TANK-binding kinase 1
Trem2: triggering receptor expressed on myeloid cells 2
UbcH5a: ubiquitin-conjugating enzyme H5a
WT: wild-type
