# Supplementary figures and images for: Peli1 impairs microglial Aβ phagocytosis through promoting C/EBPβ degradation
Source: PLoS Biol. 2020 Oct 5;18(10):e3000837. doi: 10.1371/journal.pbio.3000837 (PMC7561136; doi:10.1371/journal.pbio.3000837)

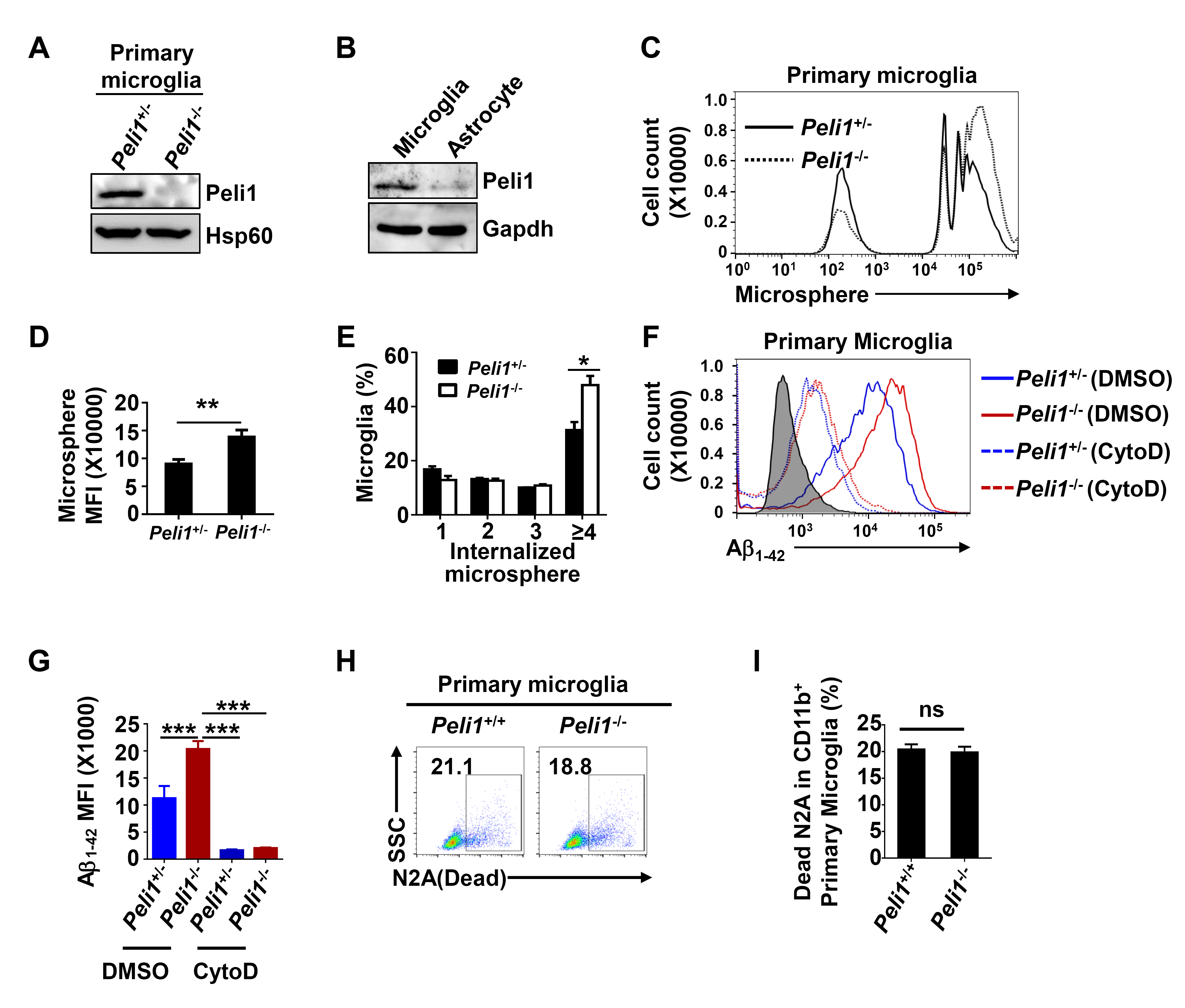

Supplement: S1 Fig — (A) Immunoblot of Peli1 and Hsp60 (loading control) in isolated heterozygous (Peli1+/−) and Peli1-deficient (Peli1−/−) murine primary microglia, showing the deleting efficiency of Peli1. (B) Immunoblot of Peli1 and Gapdh (loading control) in isolated wild-type microglia and astrocyte, showing the expression of Peli1. (C-E) Flow cytometry of the phagocytic ability for fluorescent microspheres in Peli1+/− and Peli1−/− primary microglia. The data are presented as representative histogram showing the MFI and the relative phagocytosis efficiency of microglia (C) and summary bar graphs (D, E). (F-G) Flow cytometric analysis of the phagocytic ability for Aβ1–42 in Peli1+/− and Peli1−/− primary microglia treated with DMSO or actin polymerization inhibitor Cytochalasin D (5μM) throughout Aβ1–42 incubation. The data are presented as representative histogram showing the MFI and the relative phagocytosis efficiency of microglia (F), and summary bar graphs (G). (H-I) Flow cytometric analysis of the phagocytic ability for apoptotic N2A cells in Peli1+/+ and Peli1−/− primary microglia. The data are presented as scatter plots showing the frequencies of the microglia that phagocytized with apoptotic N2A (H) and summary bar graph (I). Data with error bars represent mean ± SEM. Each panel is representative of at least 3 independent experiments. Numerical values for (D, E, G, I) are available in S1 Data. *P < 0.05, **P < 0.01, ***P < 0.001 as determined by unpaired Student t test. MFI, mean fluorescent intensity; N2A, Neuron-2A; ns, not significant. (TIF) [file pbio.3000837.s001.tif]

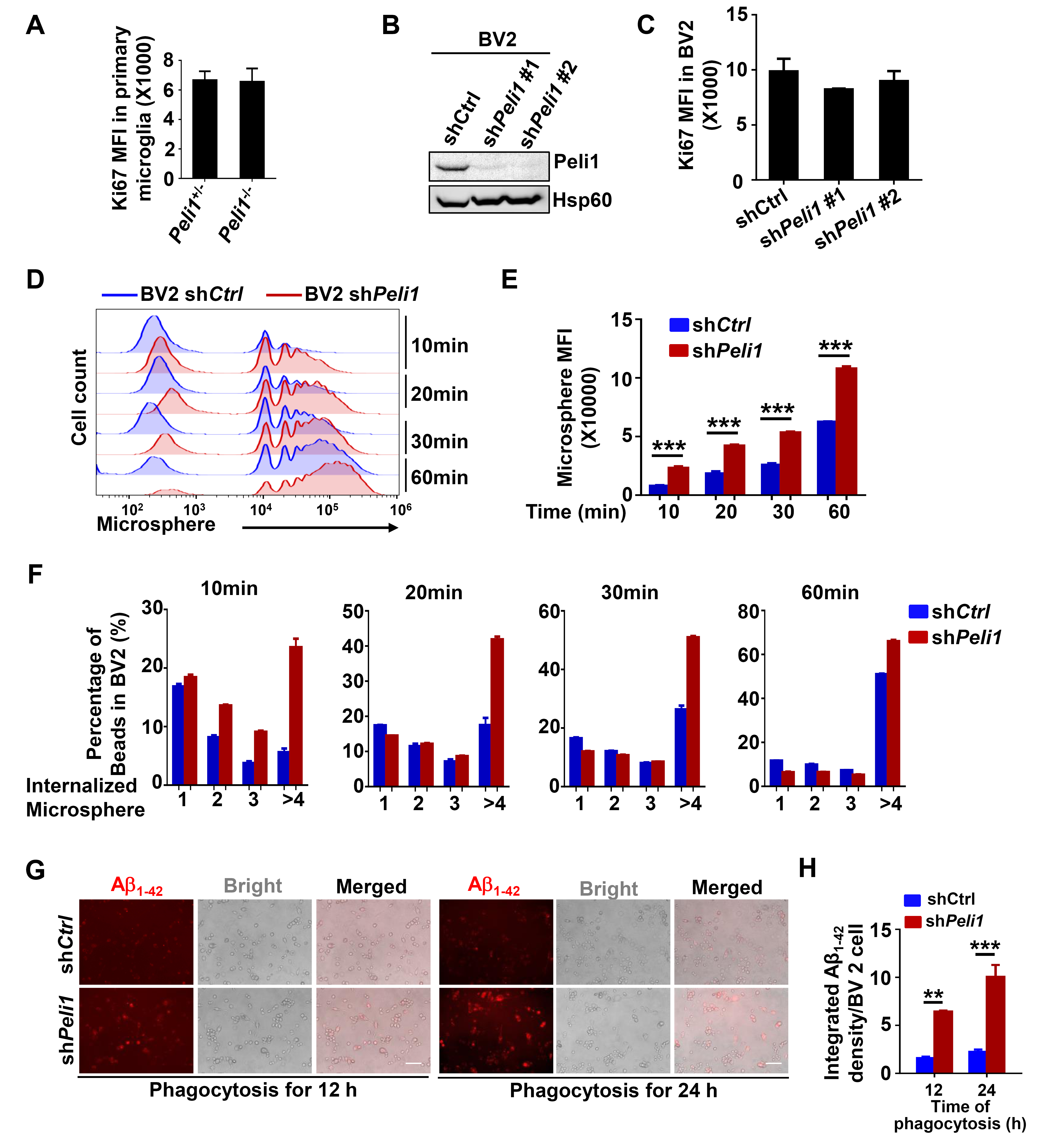

Supplement: S2 Fig — (A) Flow cytometric analysis of Ki67 expression in Peli1+/− and Peli1−/− primary microglia, the data are presented as representative histogram showing the MFI and the relative expression of Ki67 in cells. (B) Immunoblot of Peli1 and Hsp60 (loading control) in BV2 microglial cells with lentivirus encoding shRNA targeting Peli1 or control, showing the deleting efficiency of Peli1. (C) Flow cytometric analysis of Ki67 expression in Peli1-sufficient and Peli1-knockdown BV2 cells, the data are presented as representative histogram showing the MFI and the relative expression of Ki67 in cells. (D-F) Flow cytometry of the phagocytic ability for microspheres incubated with control or Peli1-knockdown BV2 cells for indicated times. The data are presented as representative histogram showing cellular microspheres MFI (D), and summary bar graphs showing the MFI (E) and the frequencies of cells that phagocytized with microspheres (F). (G, H) Microscopic analysis of the phagocytosis of Aβ1–42 peptide at the indicated time points in control and Peli1-knockdown BV2 cells. The data are presented as representative images (G) and summary bar graph (H). Scale bars: 100 μm. Data with error bars represent mean ± SEM. Each panel is representative of at least 3 independent experiments. Numerical values for (A, C, E, F, H) are available in S1 Data. *P < 0.05, **P < 0.01, ***P < 0.001 as determined by unpaired Student t test. **P < 0.01, ***P < 0.001 as determined by unpaired Student t test. Aβ, amyloid-β; MFI, mean fluorescent intensity; shRNA, short hairpin RNA. (TIF) [file pbio.3000837.s002.tif]

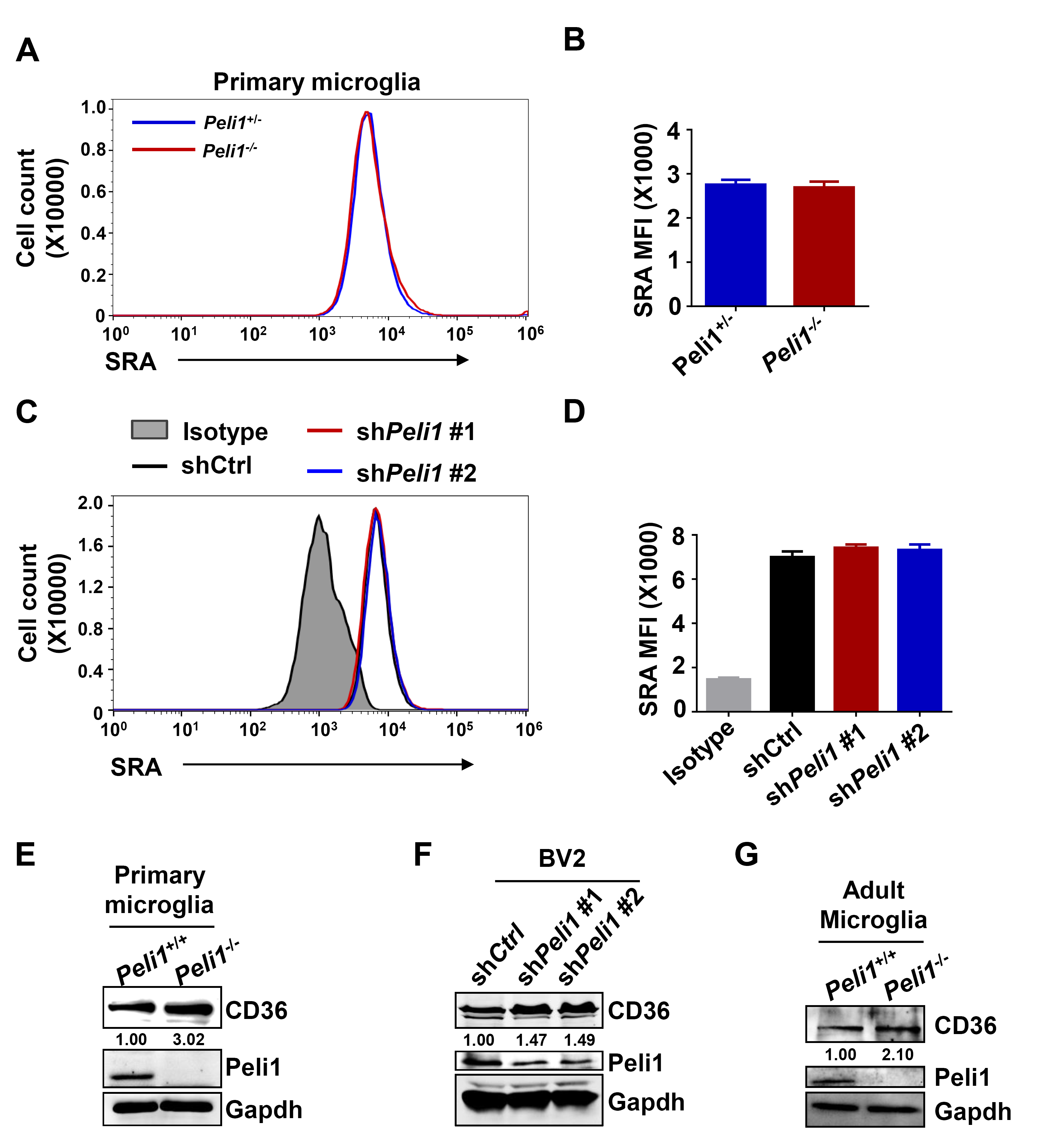

Supplement: S3 Fig — (A-D) Flow cytometry of the SRA expression on the surface of Peli1+/− and Peli1−/− microglia or control and Peli1-knockdown BV2 cells. The data are presented as representative histogram showing SRA MFI (A, C) and summary bar graphs (B, D). (E-G) Immunoblot of CD36, Peli1, and Gapdh (loading control) in Peli1-sufficient (Peli1+/+) and Peli1-deficient (Peli1−/−) primary microglia, control and Peli1-knockdown BV2 cells, and microglia isolated from Peli1+/+ and Peli1−/− adult mice. The data showed the increased expression of CD36 in Peli1-deficient cells. Data with error bars represent mean ± SEM. Each panel is representative of at least 3 independent experiments. Numerical values for (B, D) are available in S1 Data. MFI, mean fluorescent intensity; SRA, scavenger receptors class A. (TIF) [file pbio.3000837.s003.tif]

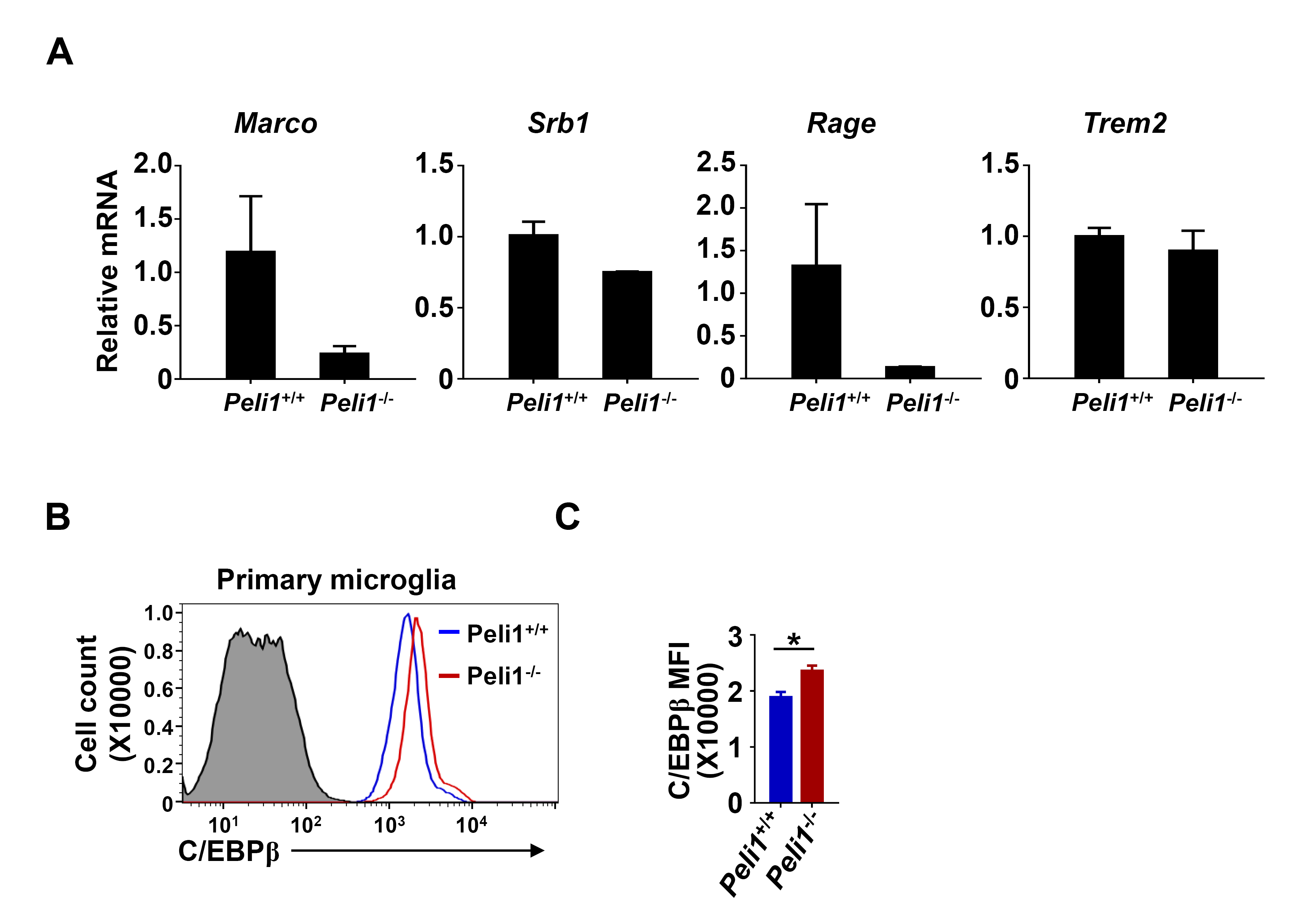

Supplement: S4 Fig — (A) Real-time qPCR analysis of Marco, Srb1, Rage, and Trem2 mRNA expressions in Peli1+/+ and Peli1−/− primary microglia. (B-C) Flow cytometric analysis of the intracellular C/EBPβ expression in Peli1+/+ and Peli1−/− primary microglia. The data are presented as representative histogram showing MFI of C/EBPβ staining (B) and summary bar graph (C). Data with error bars represent mean ± SEM. Each panel is representative of at least 3 independent experiments. Numerical values for (A, C) are available in S1 Data. *P < 0.05 as determined by unpaired Student t test. C/EBP, CCAAT/enhancer-binding protein; Marco, macrophage receptor with collagenous structure; MFI, mean fluorescent intensity; qPCR, quantitative PCR; Rage, receptor for advanced glycation end product; Srb1, scavenger receptor B-1; Trem2, triggering receptor expressed on myeloid cells 2. (TIF) [file pbio.3000837.s004.tif]

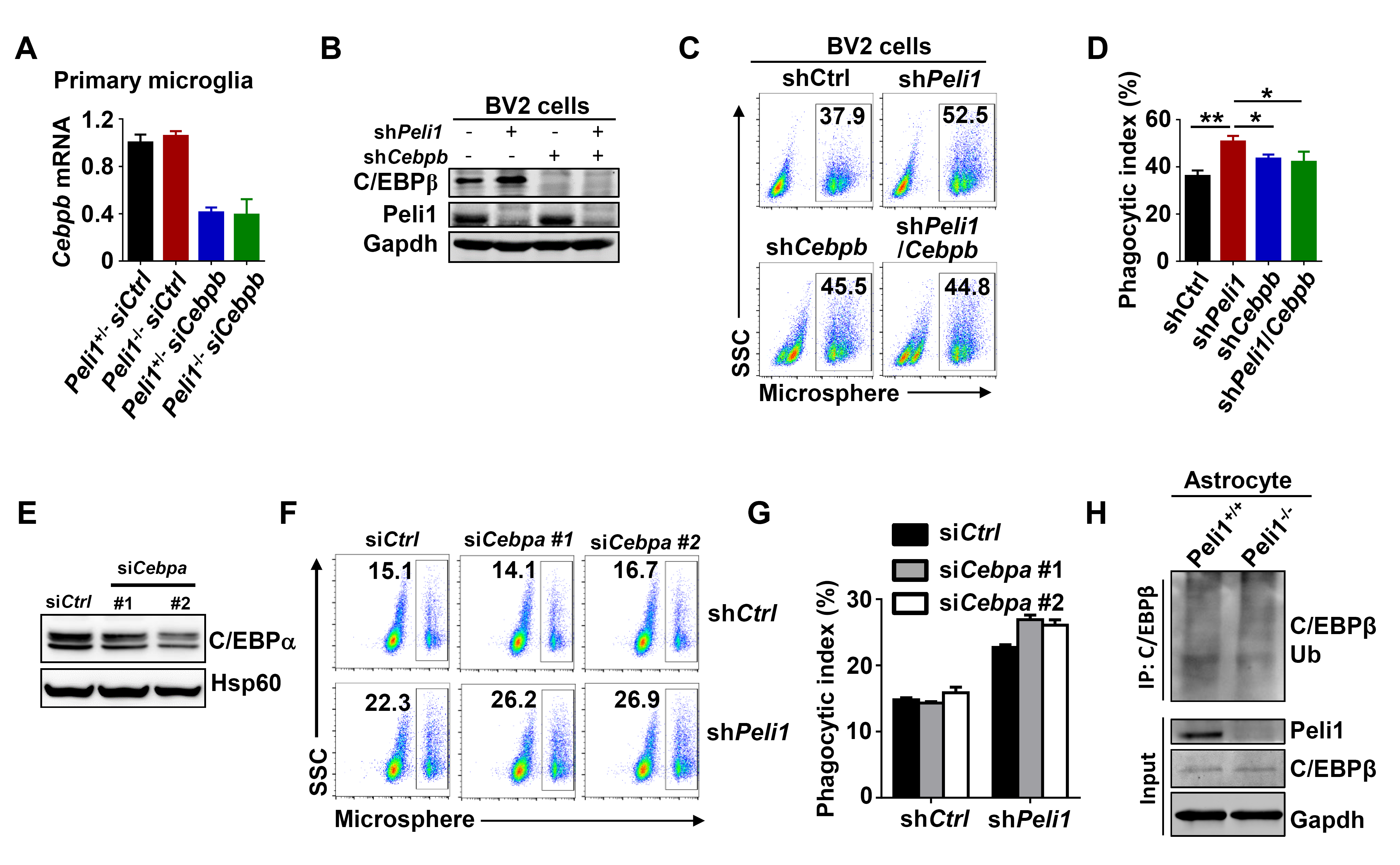

Supplement: S5 Fig — (A) qPCR analysis of Cebpb mRNA expressions in Peli1+/− and Peli1−/− primary microglia electrotransfected with siRNA targeting Cebpb or control. (B) Immunoblot of C/EBPβ, Peli1, and Gapdh (loading control) in Peli1-sufficient and Peli1-knockdown BV2 cells with or without Cebpb knockdown. (C-D) Flow cytometry of the ability of microspheres phagocytosis in Peli1-sufficient and Peli1-knockdown BV2 cells with or without Cebpb knockdown. The data are presented as representative scatter plots showing the frequencies of the cells that phagocytized with microspheres (C) and summary bar graphs (D). (E) Immunoblot of C/EBPα and Hsp60 (loading control) in BV2 cells electrotransfected with siRNA targeting Cebpa or control. The data show the knockdown efficiency of C/EBPα. (F-G) Flow cytometric analysis of the phagocytic ability for microspheres in Peli1-sufficient and Peli1-knockdown BV2 cells with or without Cebpa knockdown. The data are presented as representative scatter plots showing the frequencies of the cells that phagocytized with microspheres (F) and summary bar graphs (G). (H) Ubiquitination of endogenous C/EBPβ in Peli1+/+ and Peli1−/− primary astrocyte that were pretreated with MG132 for 4 hours, assessed by immunoblot analysis with anti-ubiquitin after immunoprecipitation with anti-C/EBPβ (top), and immunoblot analysis with Peli1, C/EBPβ, and loading control of whole-cell lysate in Peli1+/+ and Peli1−/− primary astrocyte that were not pretreated with MG132 (below). Data with error bars represent mean ± SEM. Each panel is representative of at least 3 independent experiments. Numerical values for (A, D, G) are available in S1 Data. *P < 0.05, **P < 0.01 as determined by unpaired Student t test. C/EBP, CCAAT/enhancer-binding protein; qPCR, quantitative PCR; siRNA, small interfering RNA. (TIF) [file pbio.3000837.s005.tif]

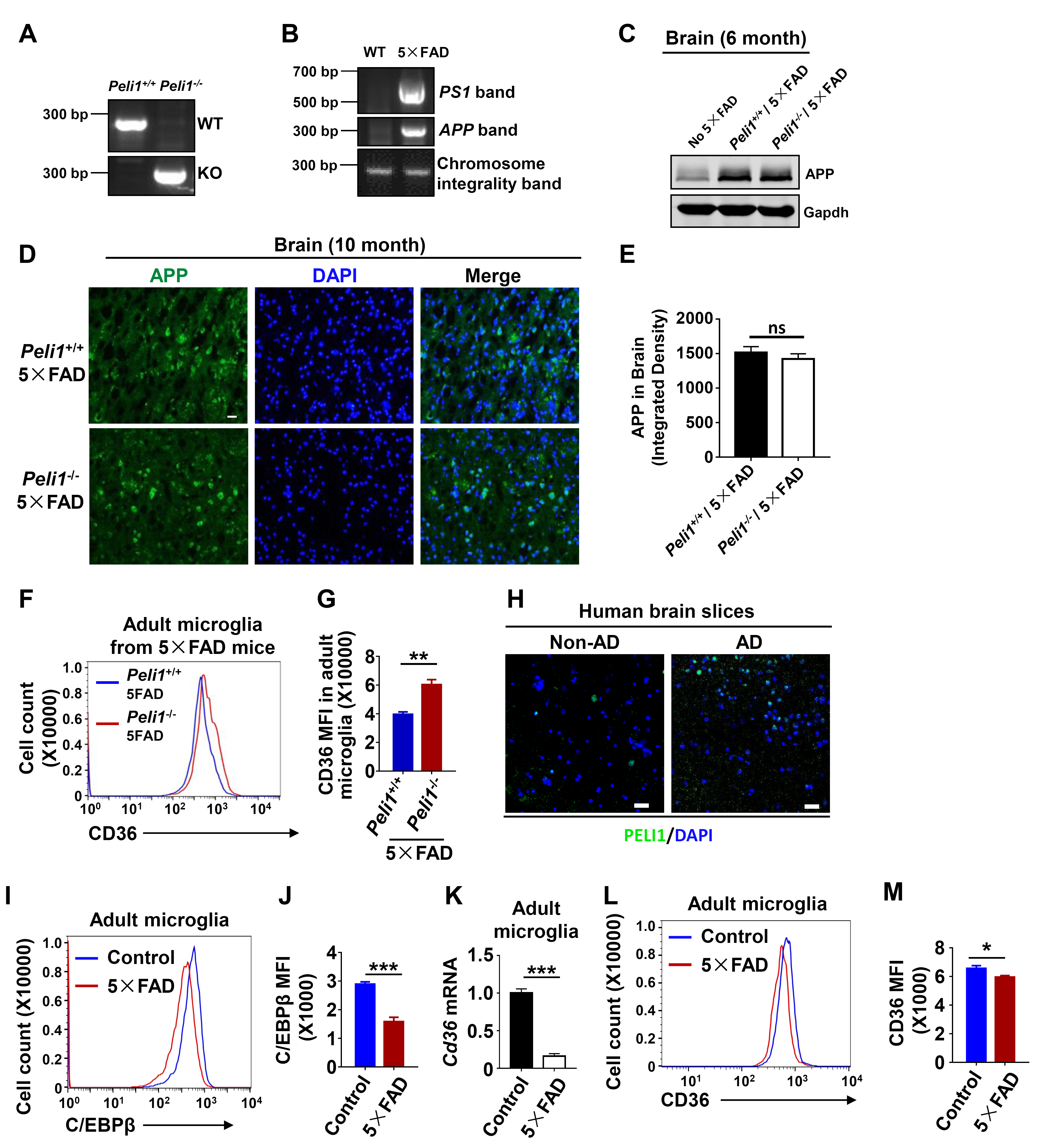

Supplement: S6 Fig — (A-B) The genotyping PCR analysis of Peli1+/+ and Peli1−/− adult mice (A) and AD-like 5×AlD transgenic mice (B). (C) Immunoblot of APP and Gapdh (loading control) in the brain tissues of 6-month-old naive, Peli1+/+ 5×FAD and Peli1−/− 5×FAD mice. (D, E) Immunofluorescent images showing APP expression in the cerebral cortex from 10-month-old Peli1+/+ 5×FAD or Peli1−/− 5×FAD male mice (n = 4 or 5 mice/group). The data are presented as representative images (D) and summary bar graph quantifying the APP expression (E). Scale bar: 20 μm. (F, G) Flow cytometry of CD36 expression on the surface of microglia isolated from age- and sex-matched aged Peli1+/+ 5×FAD or Peli1−/− 5×FAD male mice. The data are presented as representative histogram (F) and summary bar graph (G). (H) Immunofluorescent images showing PELI1 expression (green) in the brains of human AD patients and aged-matched non-AD controls. Scale bar: 30 μm. (I-J) Flow cytometry of the intracellular C/EBPβ expression in microglia isolated from 10-month-old age- and sex-matched adult naive and 5×FAD transgenic mice. The data are presented as representative histograms showing the MFI of the C/EBPβ staining (I) and summary bar graphs (J) quantifying the C/EBPβ expression. (K) qPCR analysis of Cd36 mRNA expression in microglia isolated from 10-month-old age- and sex-matched adult naive and 5×FAD transgenic mice. (L-M) Flow cytometry of the surface CD36 expression in microglia isolated from 10-month-old age- and sex-matched adult naive and 5×FAD transgenic mice. The data are presented as representative histograms (L) and summary bar graphs (M). Data with error bars represent mean ± SEM. Each panel is representative of at least 3 independent experiments. Numerical values for (E, G, J, K, M) are available in S1 Data. *P < 0.05, **P < 0.01, ***P < 0.001 as determined by unpaired Student t test. AD, Alzheimer’s disease; APP, amyloid-beta precursor protein; C/EBP, CCAAT/enhancer-binding protein; MFI, mean fluorescent intensit [file pbio.3000837.s006.tif]
