# Supplementary material for: Peli1 impairs microglial Aβ phagocytosis through promoting C/EBPβ degradation
Source: PLoS Biol. 2020 Oct 5;18(10):e3000837. doi: 10.1371/journal.pbio.3000837 (PMC7561136; doi:10.1371/journal.pbio.3000837)
Supplement: S1 Table — ChIP, chromatin immunoprecipitation; qPCR, quantitative PCR. (DOCX) [file pbio.3000837.s007.docx]

**S1 Table. Primers used for real-time QPCR or ChIP-QPCR.**

| **Genes** | **Forward primers (5′-3′)** | **Reverse primers (5′-3′)** |
| --- | --- | --- |
| *Cd36* | GGACATTGAGATTCTTTTCCTCTG | GCAAAGGCATTGGCTGGAAGAAC |
| *Cebpb* | CAACCTGGAGACGCAGCACAAG | GCTTGAACAAGTTCCGCAGGGT |
| *Peli1* | CCTTGTCCATGTAAGTTTCTC | CAGAGTTCAGAAGTCTGGAACT |
| *Marco* | ATGGCACCAAGGGAGACAAAGG | GCCTGGTTTTCCAGCATCACCT |
| *Srb1* | ACACCCGAATCCTCGCTGGAAT | CCGTTGGCAAACAGAGTATCGG |
| *Rage* | GCCACTGGAATTGTCGATGAGG | GCTGTGAGTTCAGAGGCAGGAT |
| *Trem2* | CTACCAGTGTCAGAGTCTCCGA | CCTCGAAACTCGATGACTCCTC |
| *Actin* | CGTGAAAAGATGACCCAGATCA | CACAGCCTGGATGGCTACGT |
| *Cd36*  (ChIP-qPCR) | GTTGATTGACAAGAGTTTCCAATTGTT | ATTAGGGAACTGATTGGTCTGTCCCA |
