# Supplementary material for: Peli1 impairs microglial Aβ phagocytosis through promoting C/EBPβ degradation
Source: PLoS Biol. 2020 Oct 5;18(10):e3000837. doi: 10.1371/journal.pbio.3000837 (PMC7561136; doi:10.1371/journal.pbio.3000837)
Supplement: S1 Raw images — (PDF) [file pbio.3000837.s009.pdf]

Fig 1M

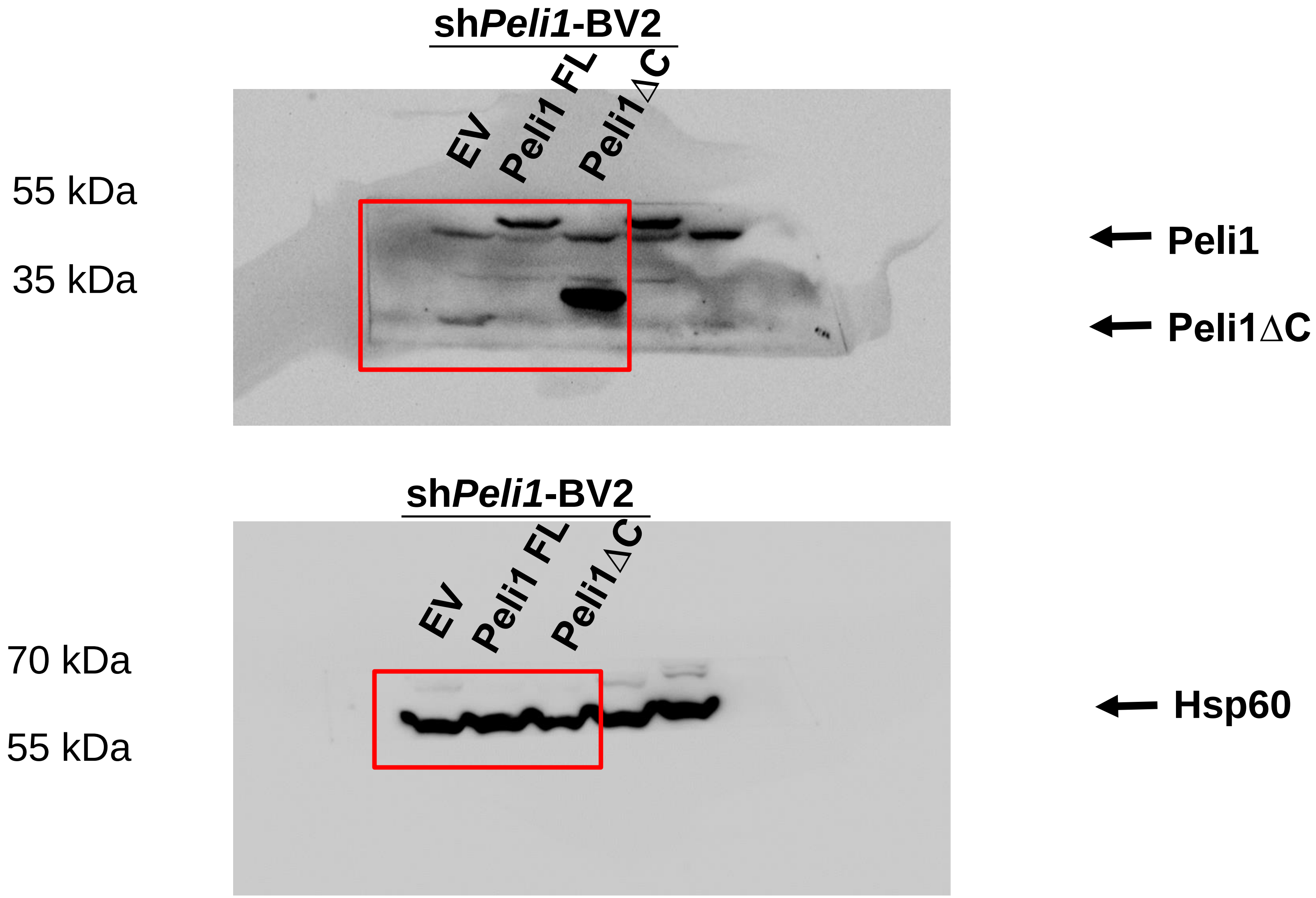
shPeli1-BV2
EV
Peli1 FL
Peli1ΔC
55 kDa
35 kDa
Peli1
Peli1ΔC
shPeli1-BV2
EV
Peli1 FL
Peli1ΔC
70 kDa
55 kDa
Hsp60

Fig 2I

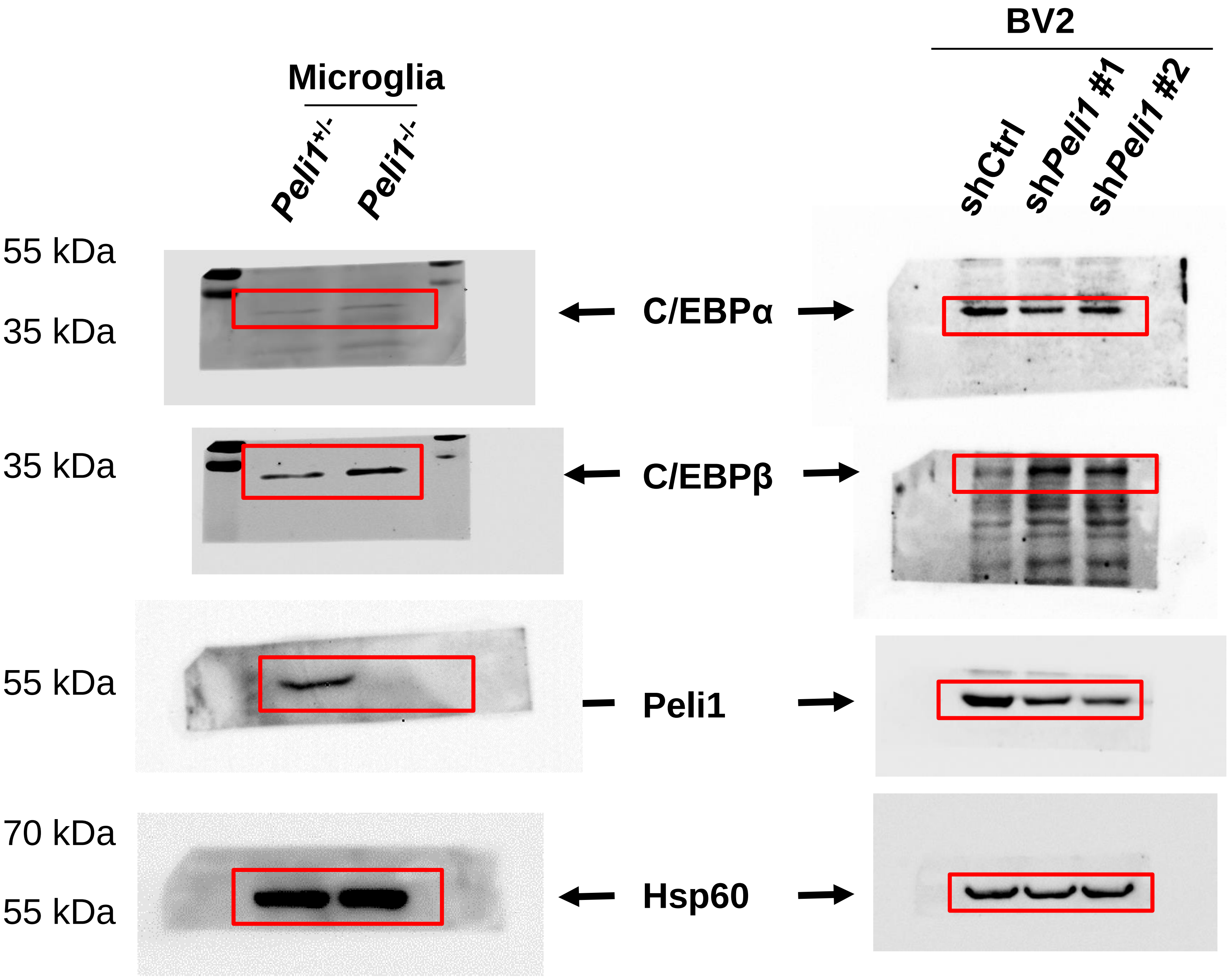

Microglia
Peli1+/-
Peli1-/-
BV2
shCtrl
shPeli1 #1
shPeli1 #2
55 kDa
35 kDa
C/EBPα
35 kDa
C/EBPβ
55 kDa
Peli1
70 kDa
55 kDa
Hsp60

Fig 3B

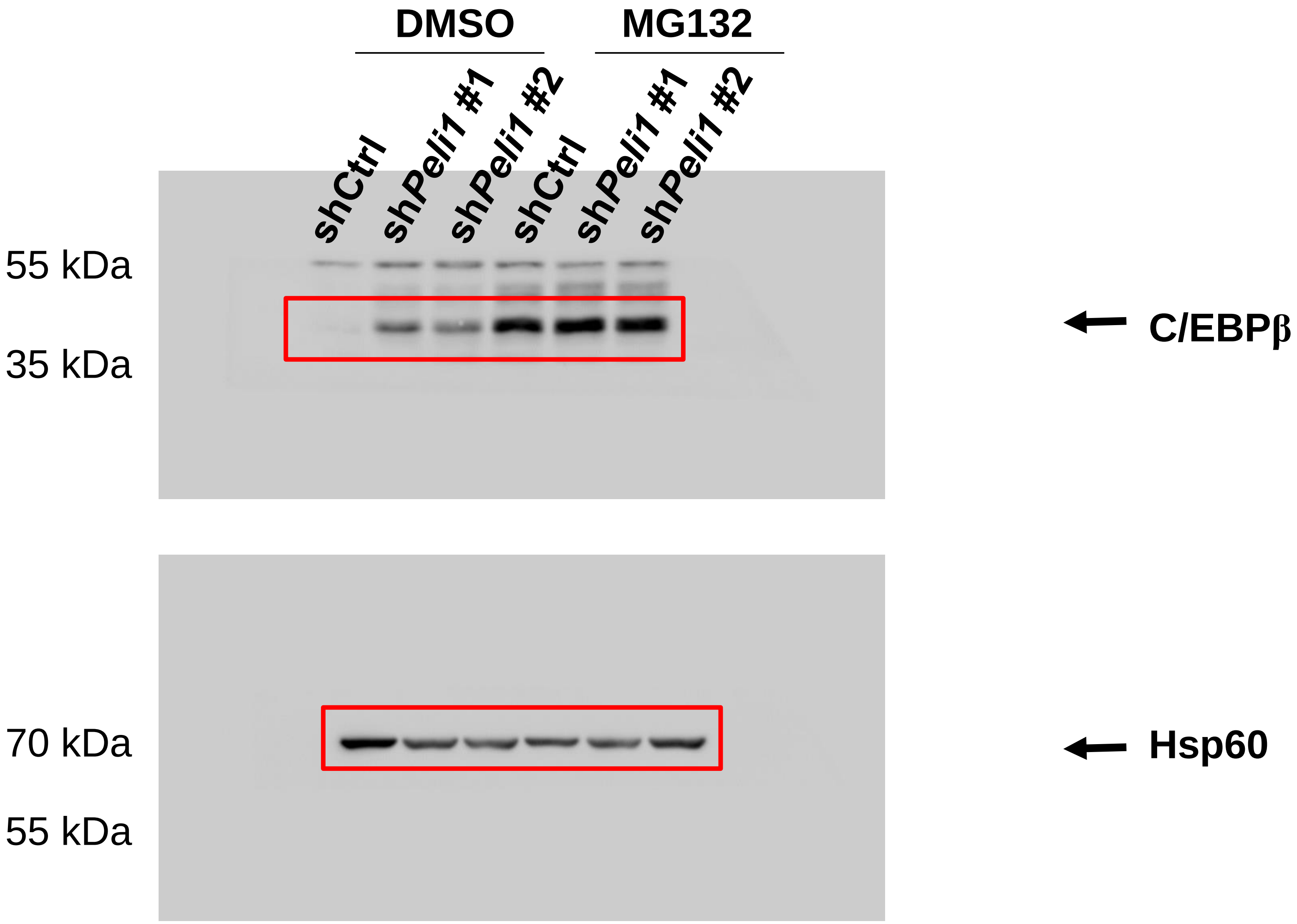

DMSO
MG132
shCtrl
shPeli1 #1
shPeli1 #2
shCtrl
shPeli1 #1
shPeli1 #2
55 kDa
35 kDa
C/EBPβ
70 kDa
55 kDa
Hsp60

Fig 3D

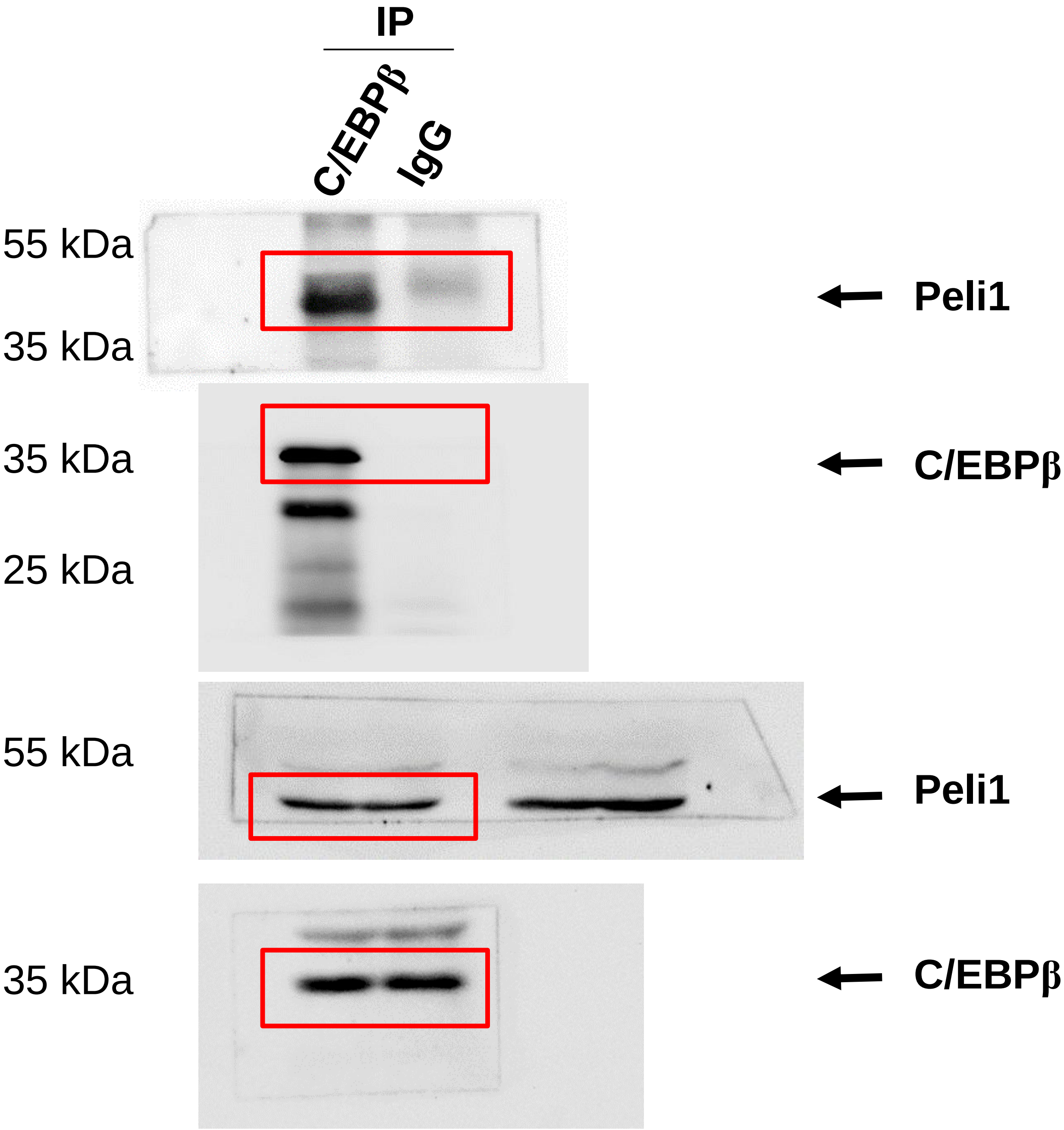

IP
C/EBPβ
IgG
55 kDa
35 kDa
Peli1
35 kDa
25 kDa
C/EBPβ
55 kDa
Peli1
35 kDa
C/EBPβ

Fig 3E

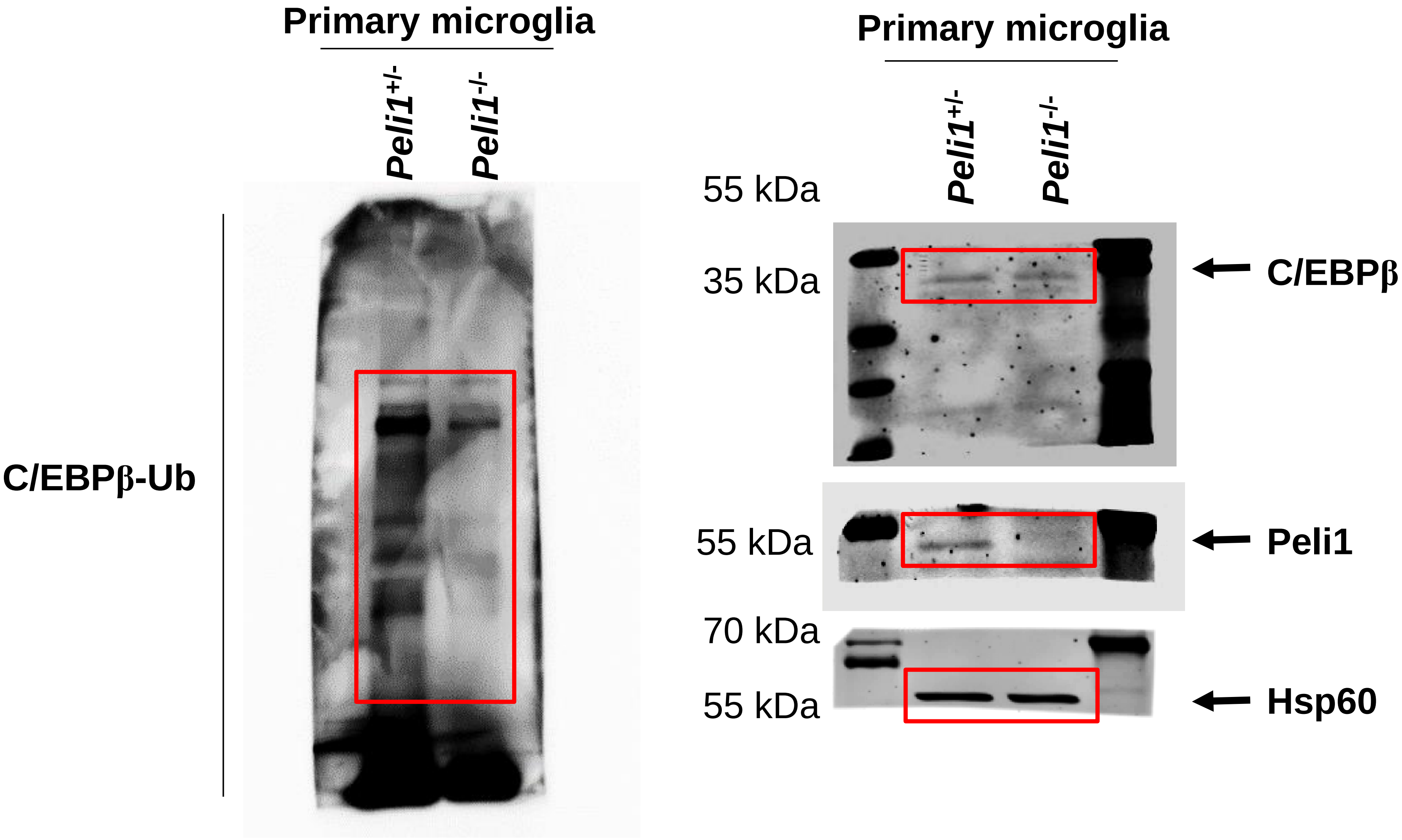

Primary microglia
Peli1+/-
Peli1-/-
C/EBPβ-Ub
Primary microglia
Peli1+/-
Peli1-/-
55 kDa
35 kDa
C/EBPβ
55 kDa
Peli1
70 kDa
55 kDa
Hsp60

Fig 3F

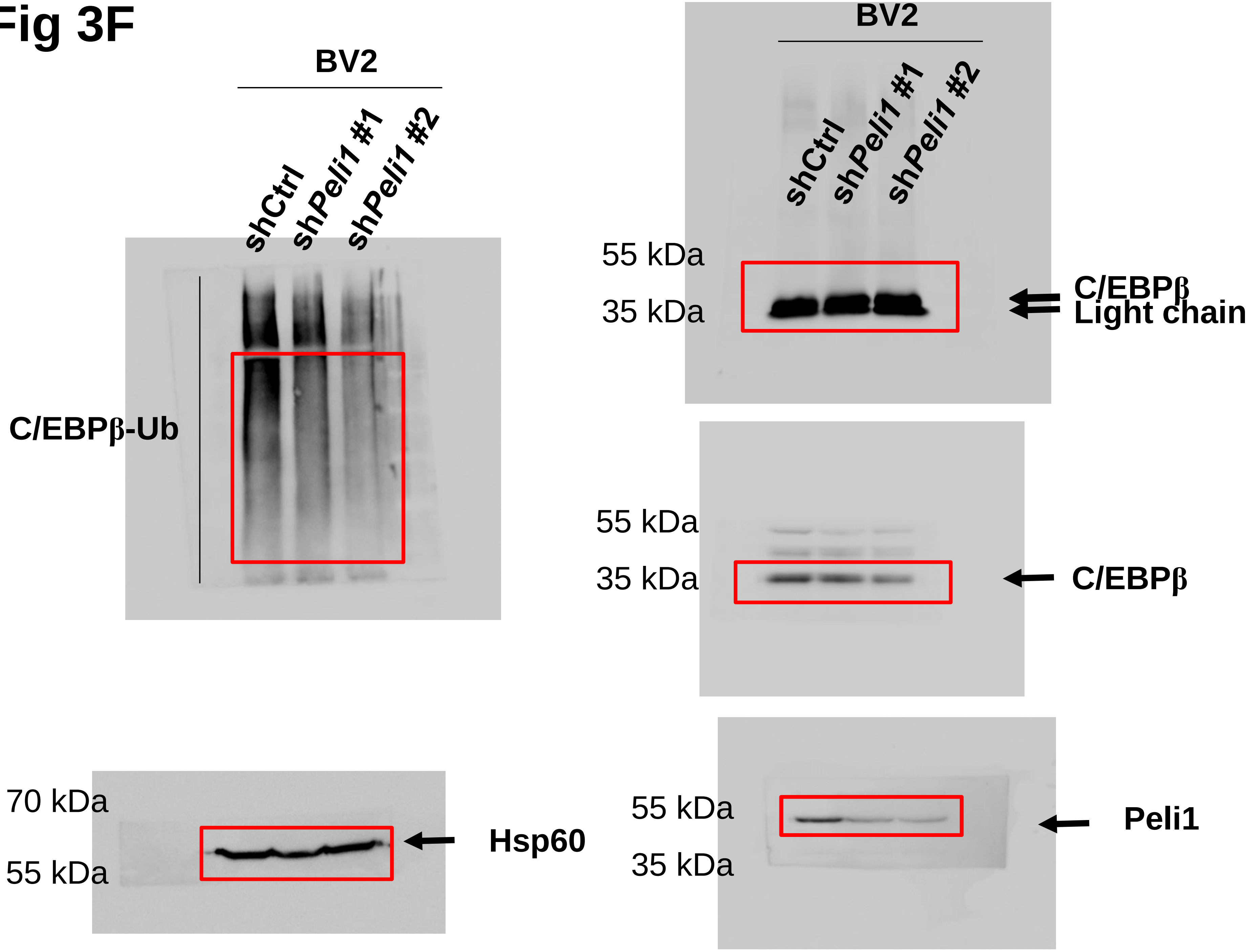
BV2
shCtrl
shPeli1 #1
shPeli1 #2
C/EBPβ-Ub
BV2
shCtrl
shPeli1 #1
shPeli1 #2
55 kDa
35 kDa
C/EBPβ
Light chain
55 kDa
35 kDa
C/EBPβ
70 kDa
55 kDa
Hsp60
55 kDa
35 kDa
Peli1

Fig 3G

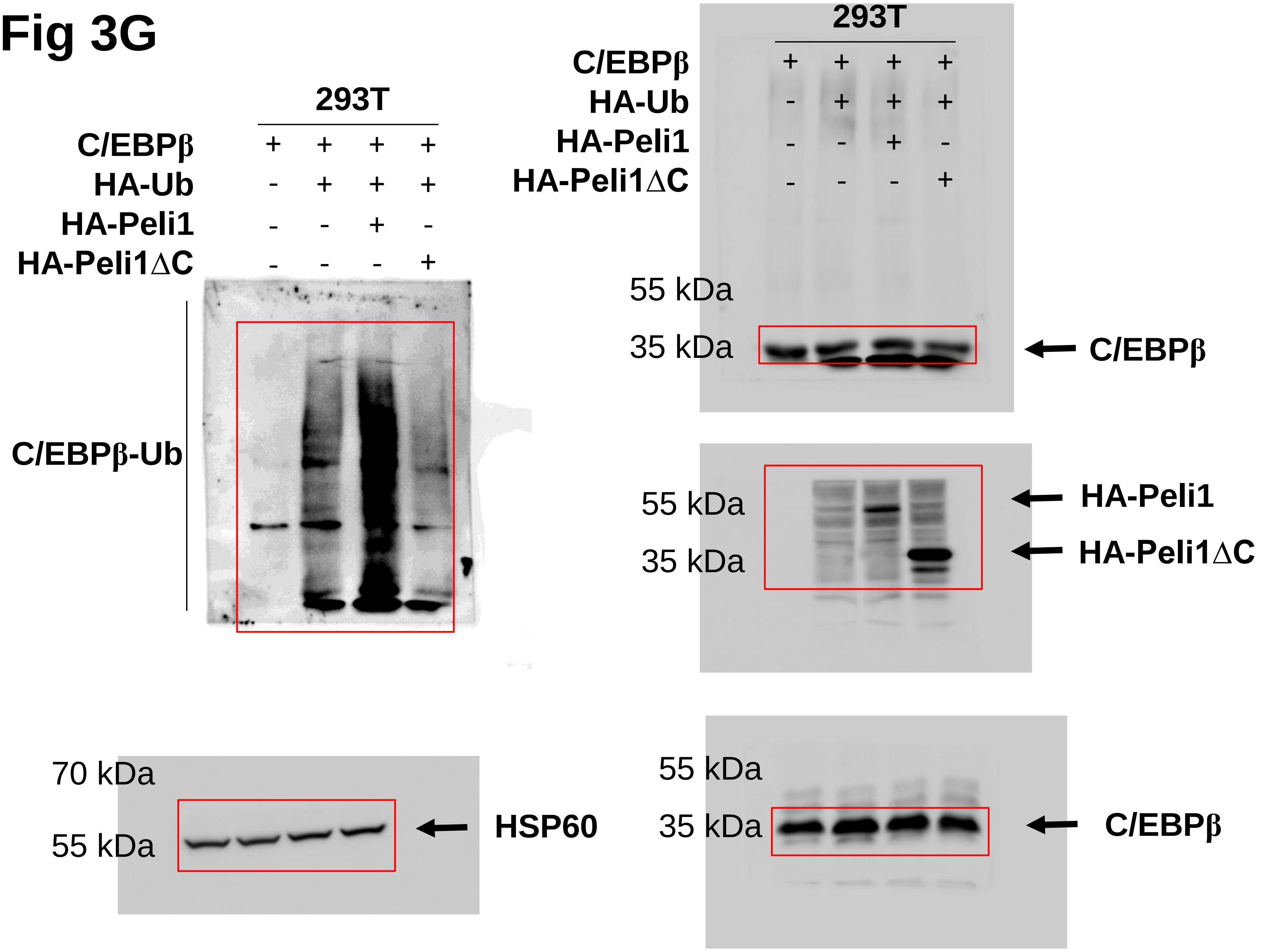

293T
C/EBPβ + + + +
HA-Ub - + + +
HA-Peli1 - - + -
HA-Peli1ΔC - - - +
C/EBPβ-Ub
55 kDa
35 kDa
C/EBPβ
HA-Peli1
HA-Peli1ΔC
70 kDa
HSP60

Fig 3H

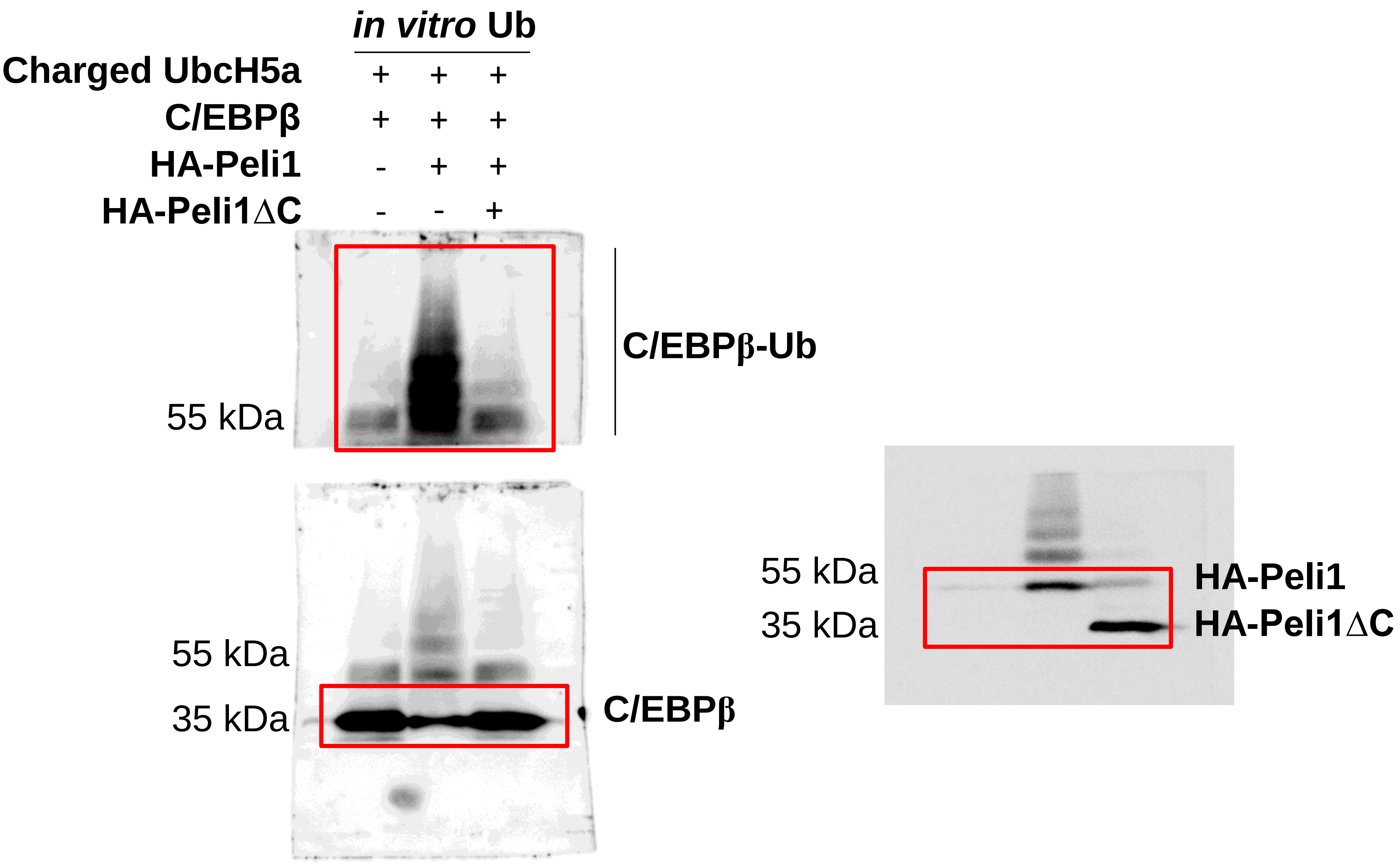

in vitro Ub
Charged UbcH5a + + +
C/EBPβ + + +
HA-Peli1 - + +
HA-Peli1ΔC - - +
55 kDa
C/EBPβ-Ub
55 kDa
35 kDa
C/EBPβ
55 kDa
35 kDa
HA-Peli1
HA-Peli1ΔC

S1A Fig

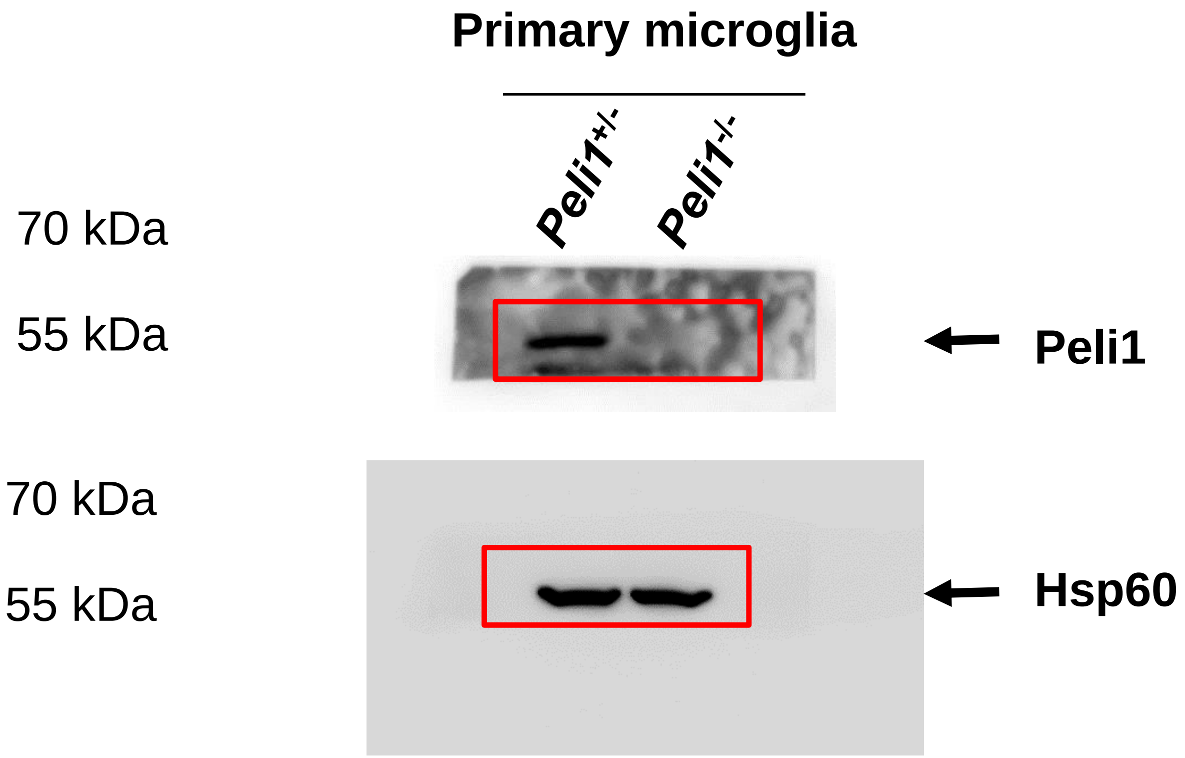
Primary microglia
Peli1+/-
Peli1-/-
70 kDa
55 kDa
Peli1
70 kDa
55 kDa
Hsp60

S1B Fig

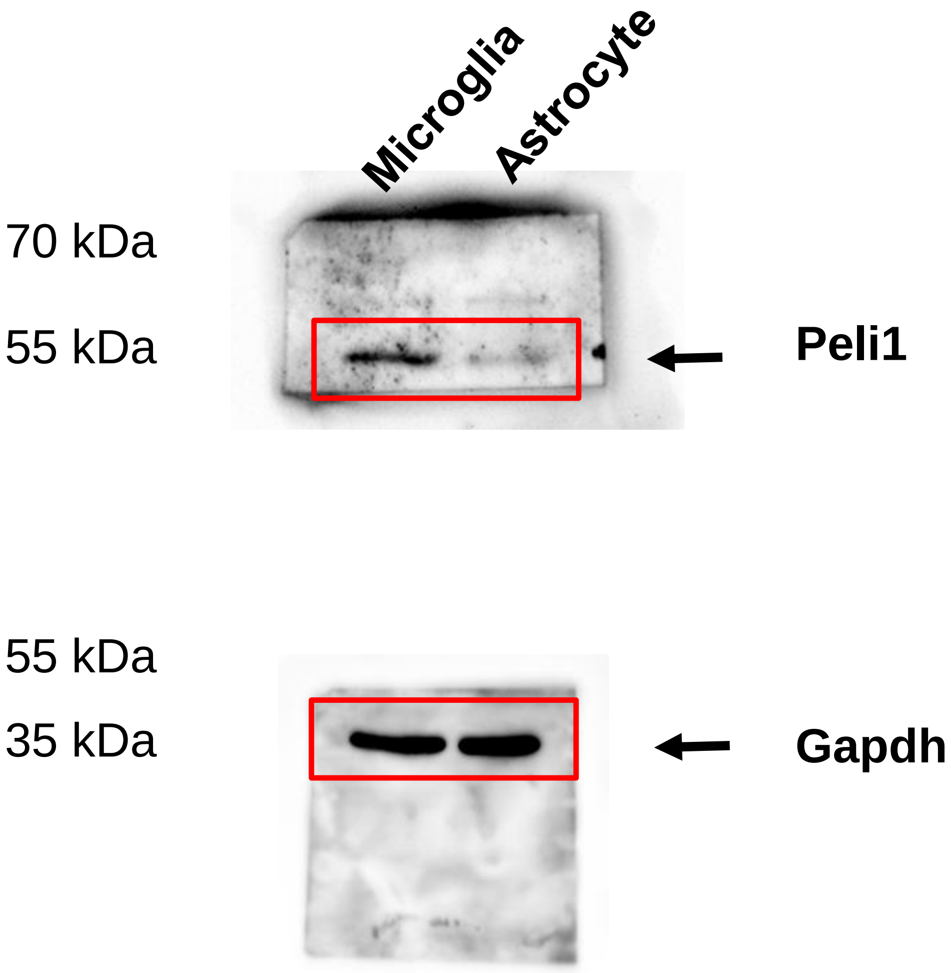

S2B Fig

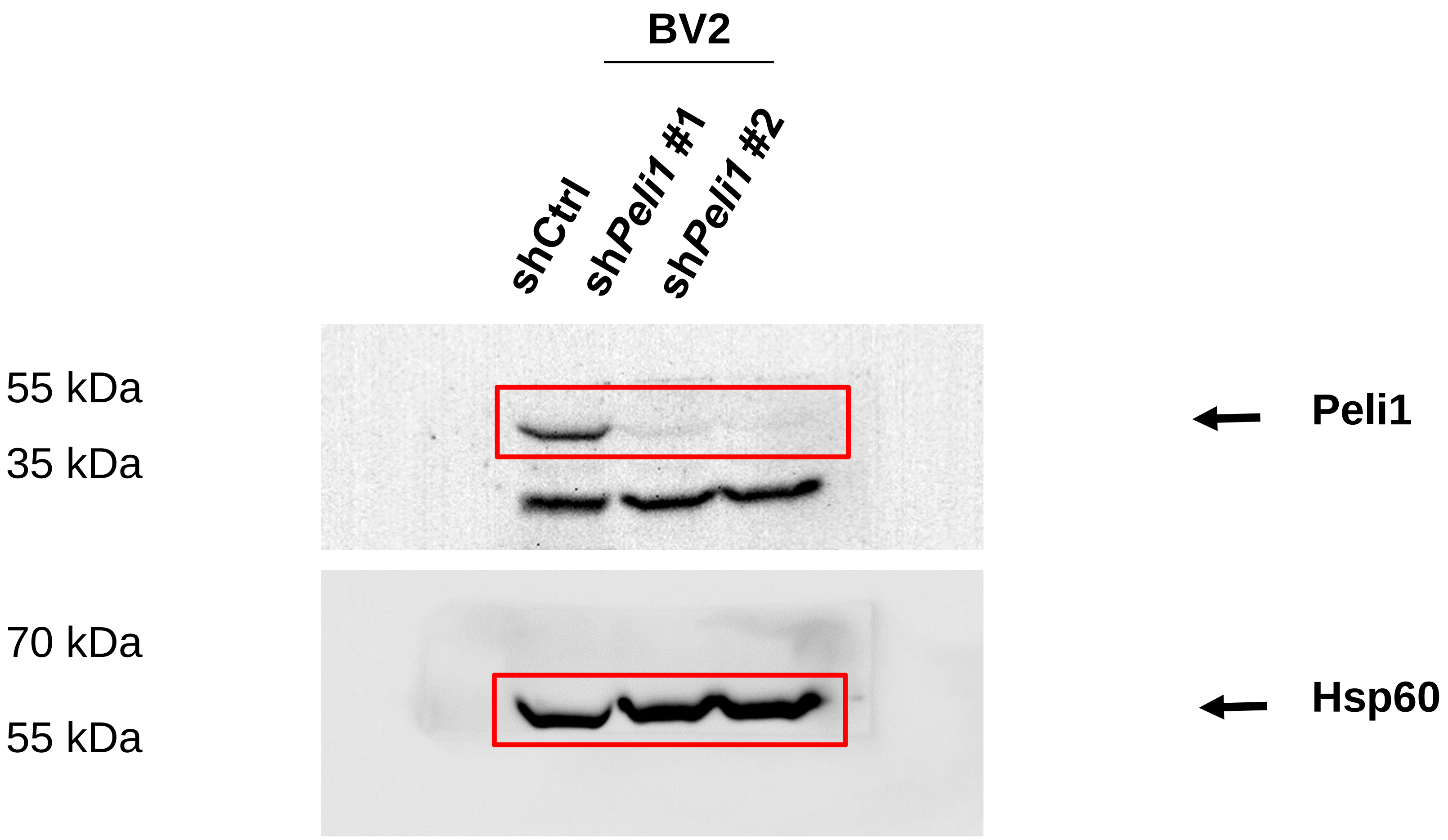
BV2
shCtrl
shPeli1 #1
shPeli1 #2
55 kDa
35 kDa
Peli1
70 kDa
55 kDa
Hsp60

S3E Fig

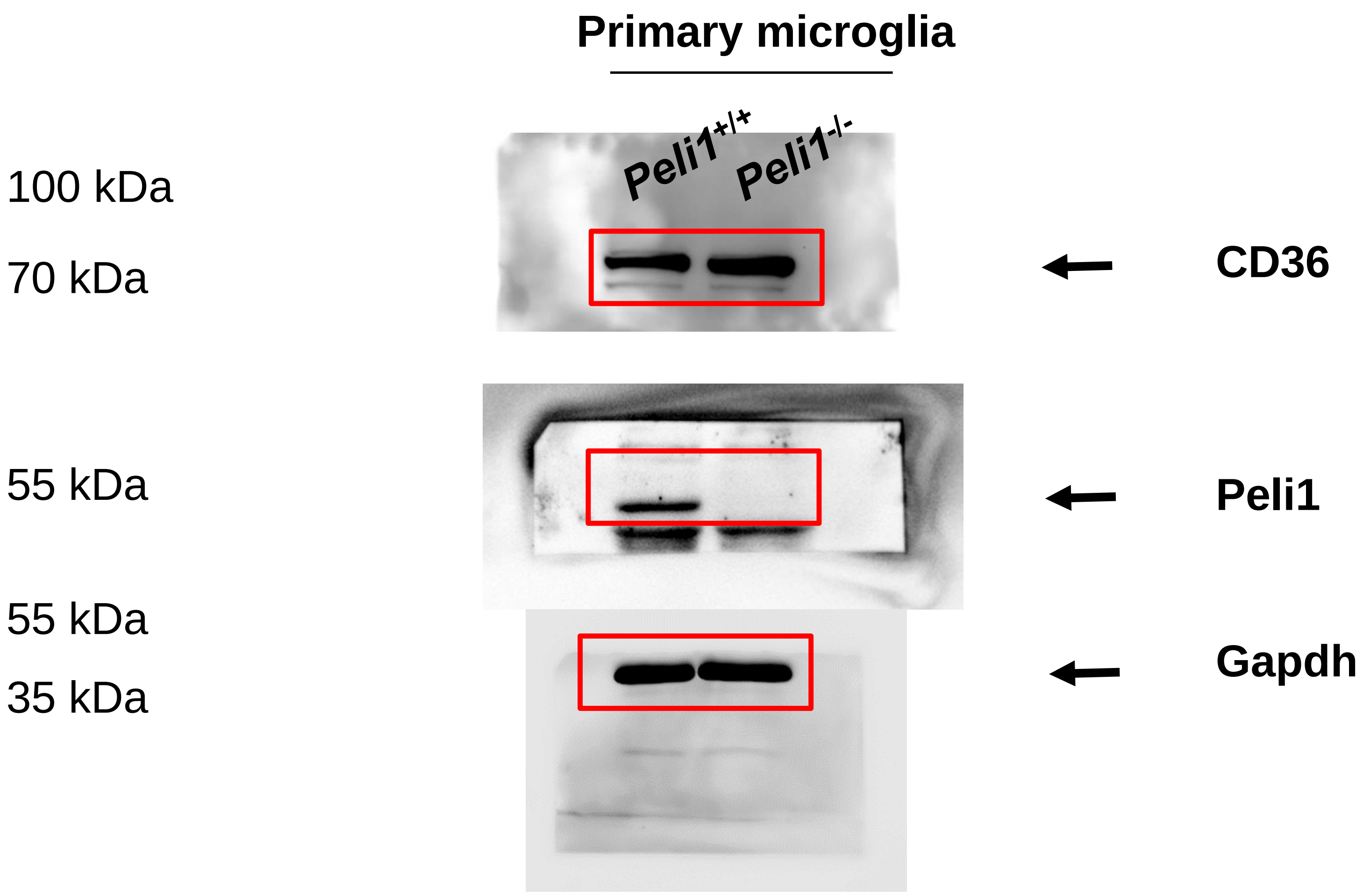

Primary microglia
Peli1+/+
Peli1-/-
100 kDa
70 kDa
55 kDa
55 kDa
35 kDa
CD36
Peli1
Gapdh

S3F Fig

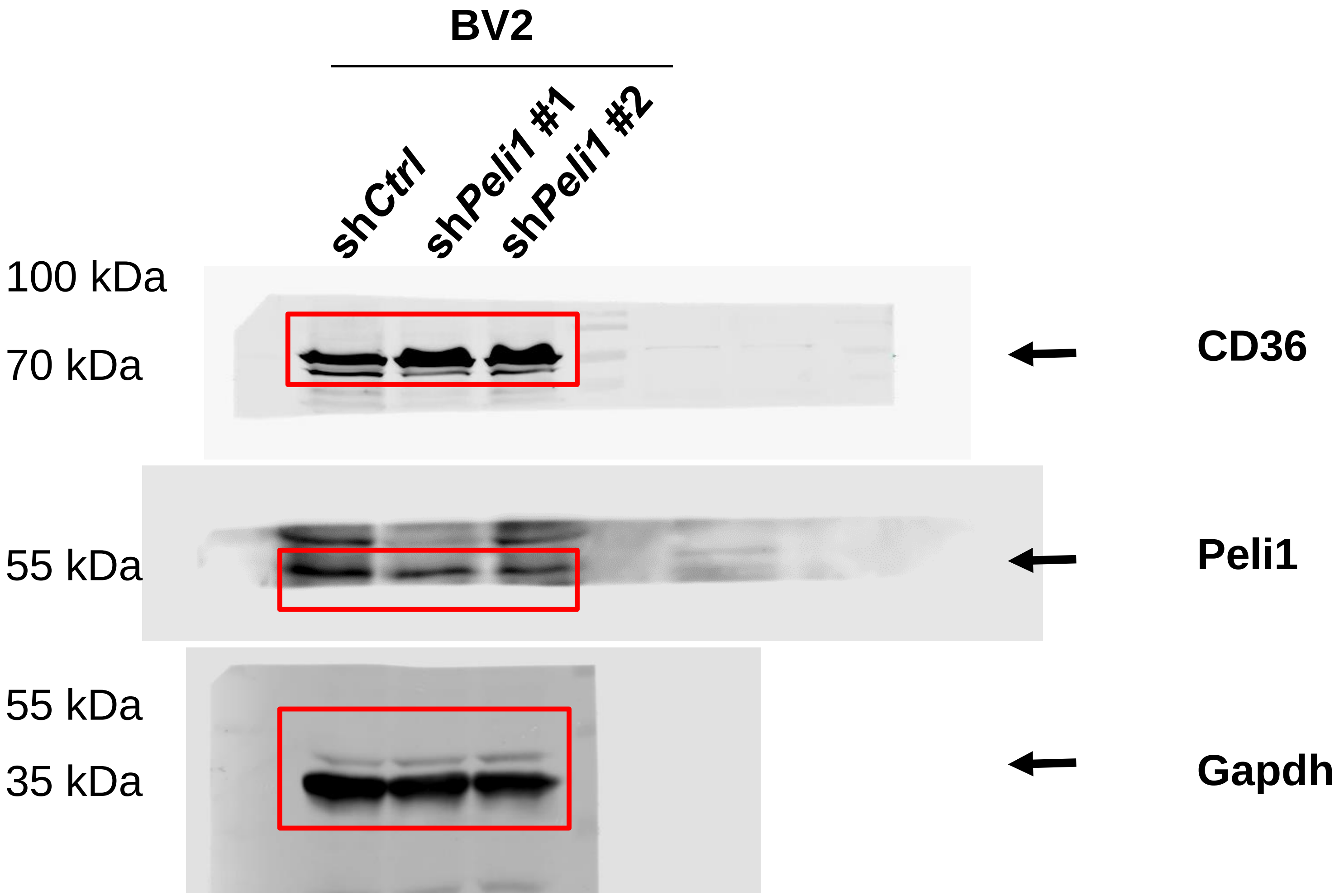
BV2
shCtrl
shPeli1 #1
shPeli1 #2
100 kDa
70 kDa
CD36
55 kDa
Peli1
55 kDa
35 kDa
Gapdh

S3G Fig

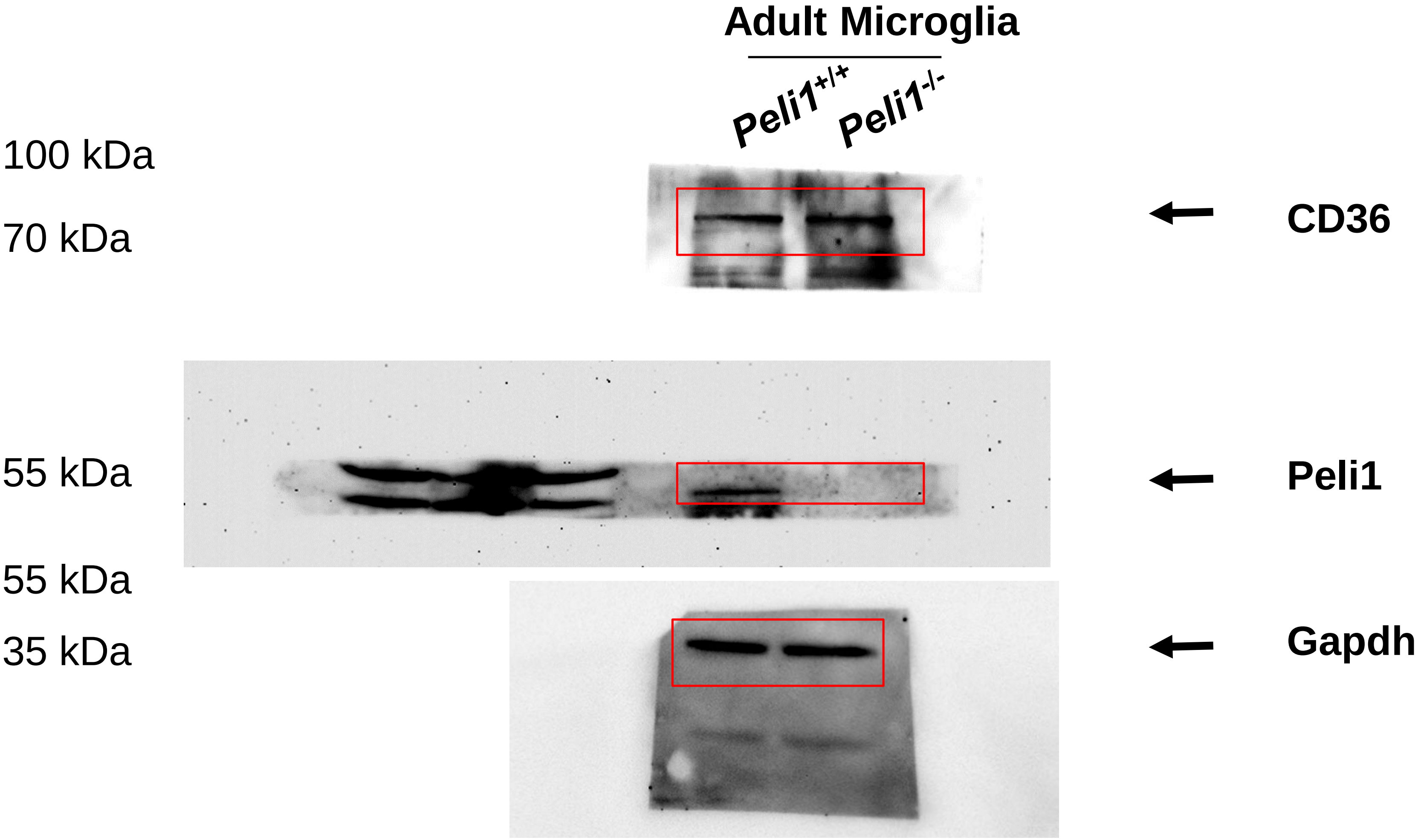
Adult Microglia
Peli1+/+
Peli1-/-
100 kDa
70 kDa
55 kDa
55 kDa
35 kDa
CD36
Peli1
Gapdh

S5B Fig

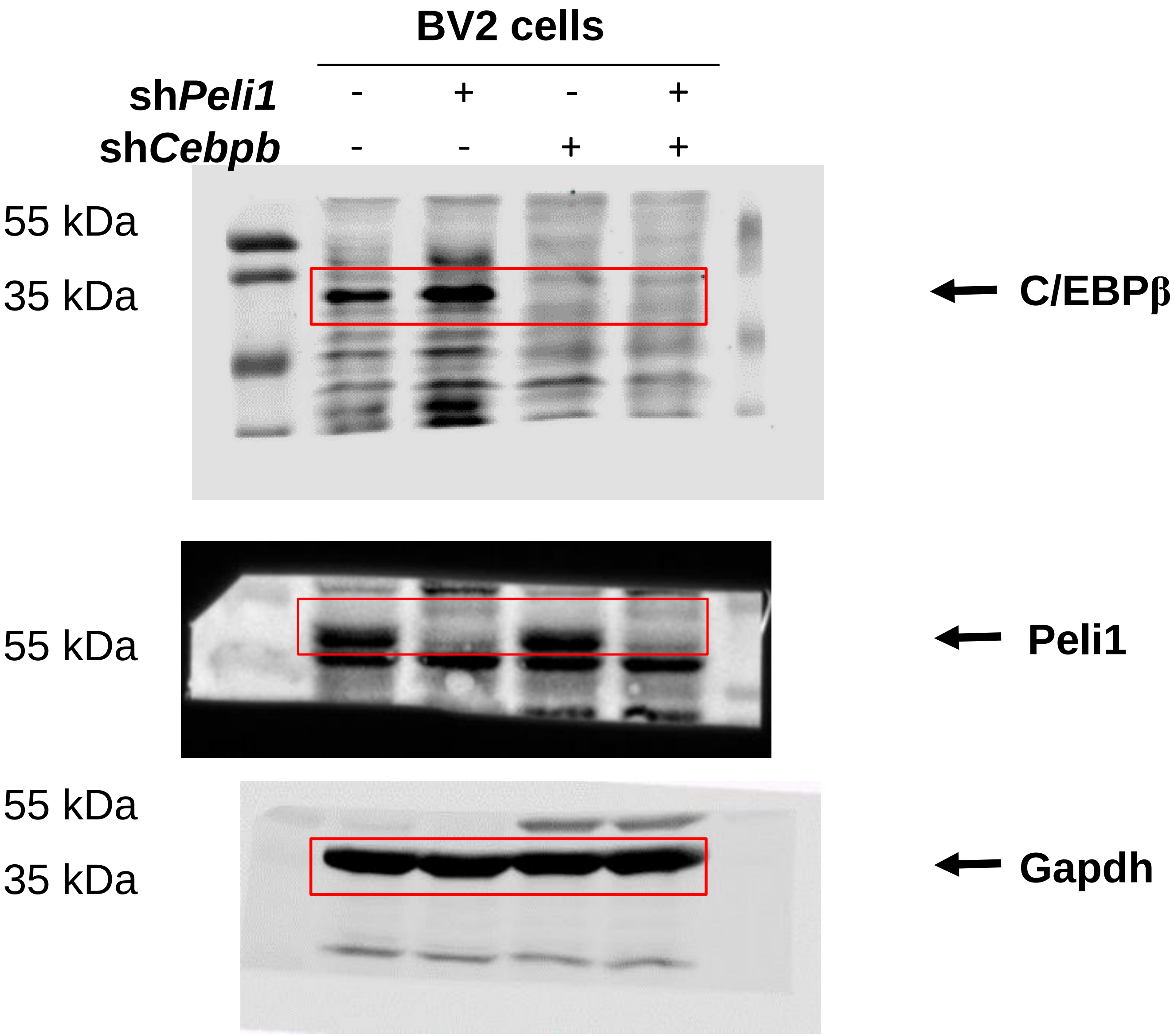

BV2 cells
shPeli1 - + - +
shCebpb - - + +
55 kDa
35 kDa
C/EBPβ
55 kDa
Peli1
55 kDa
35 kDa
Gapdh

S5E Fig

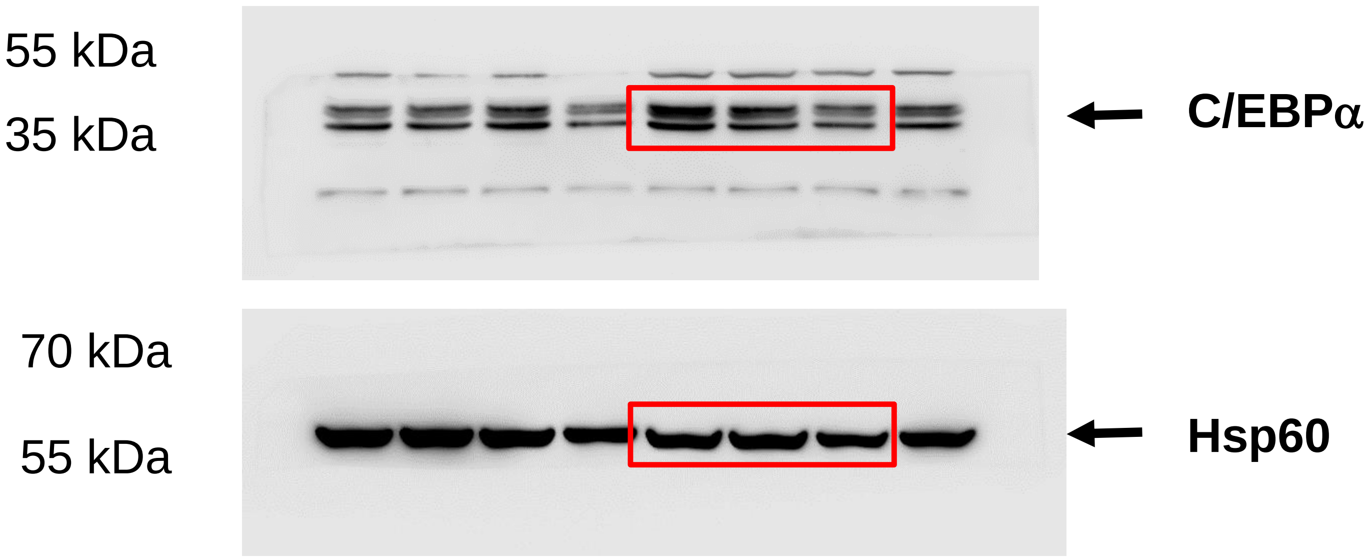

S5H Fig

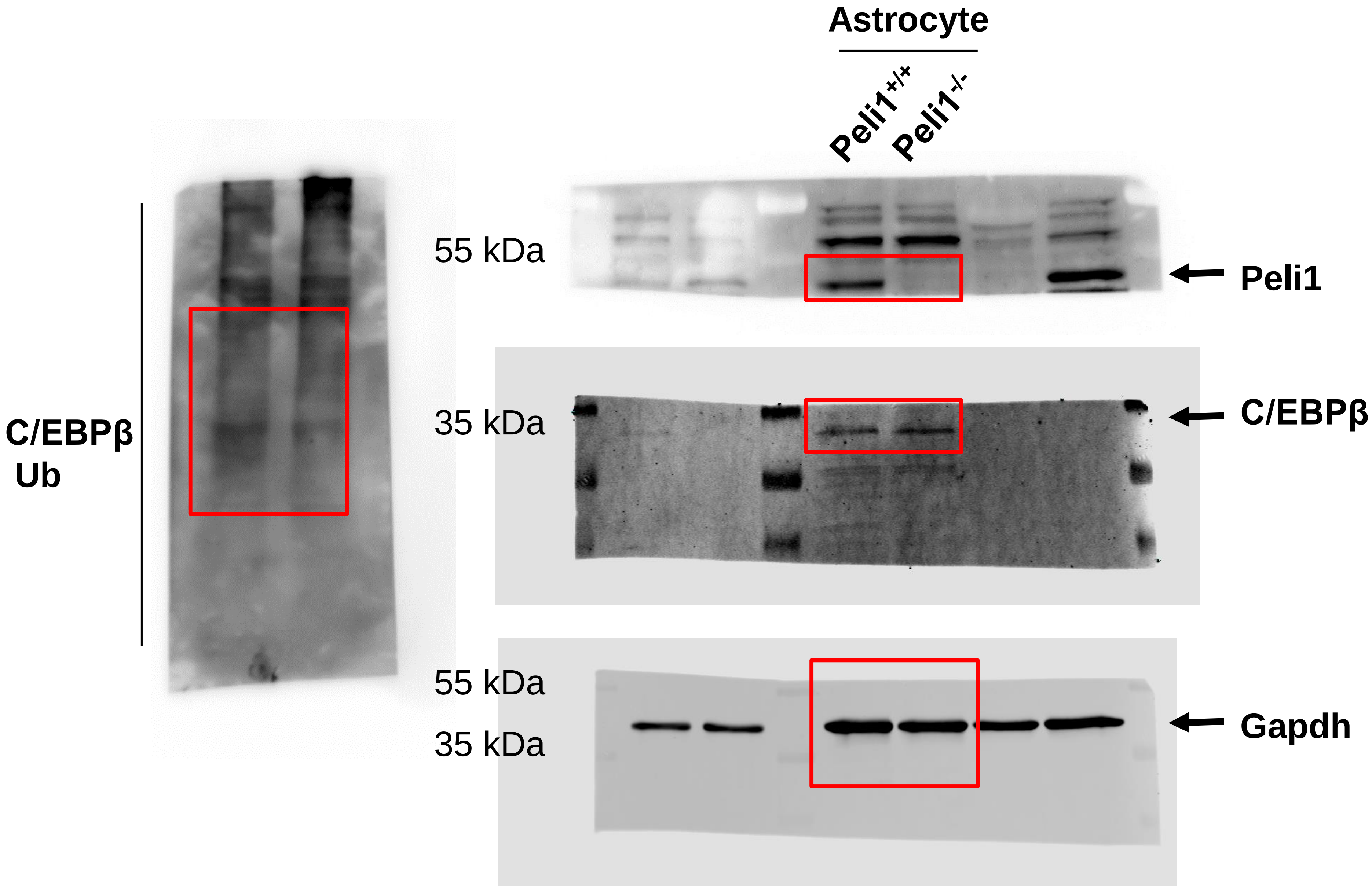

Astrocyte
Peli1+/+
Peli1-/-
C/EBPβ
Ub
55 kDa
35 kDa
55 kDa
35 kDa
Peli1
C/EBPβ
Gapdh

S6A Fig

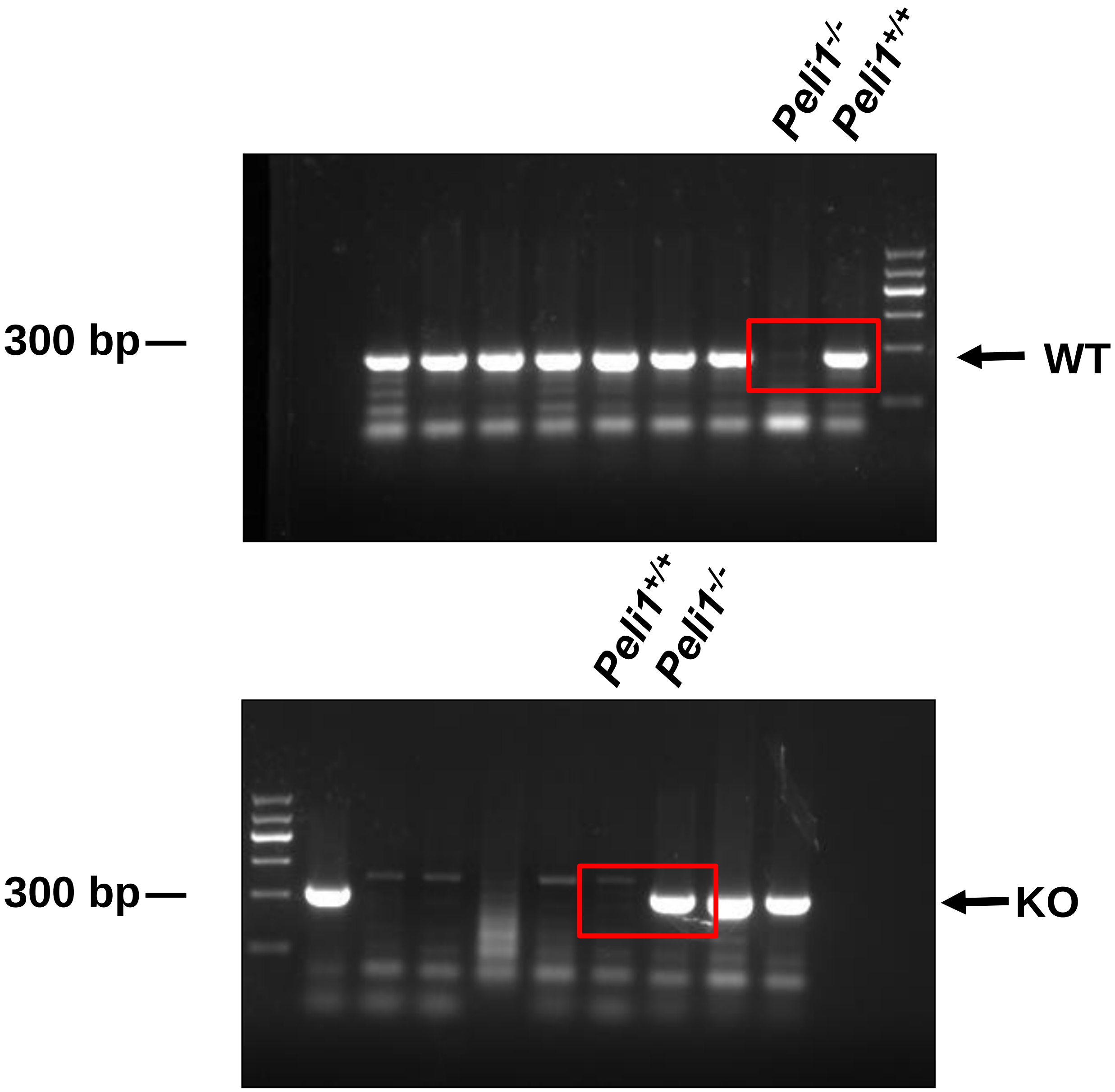

S6B Fig

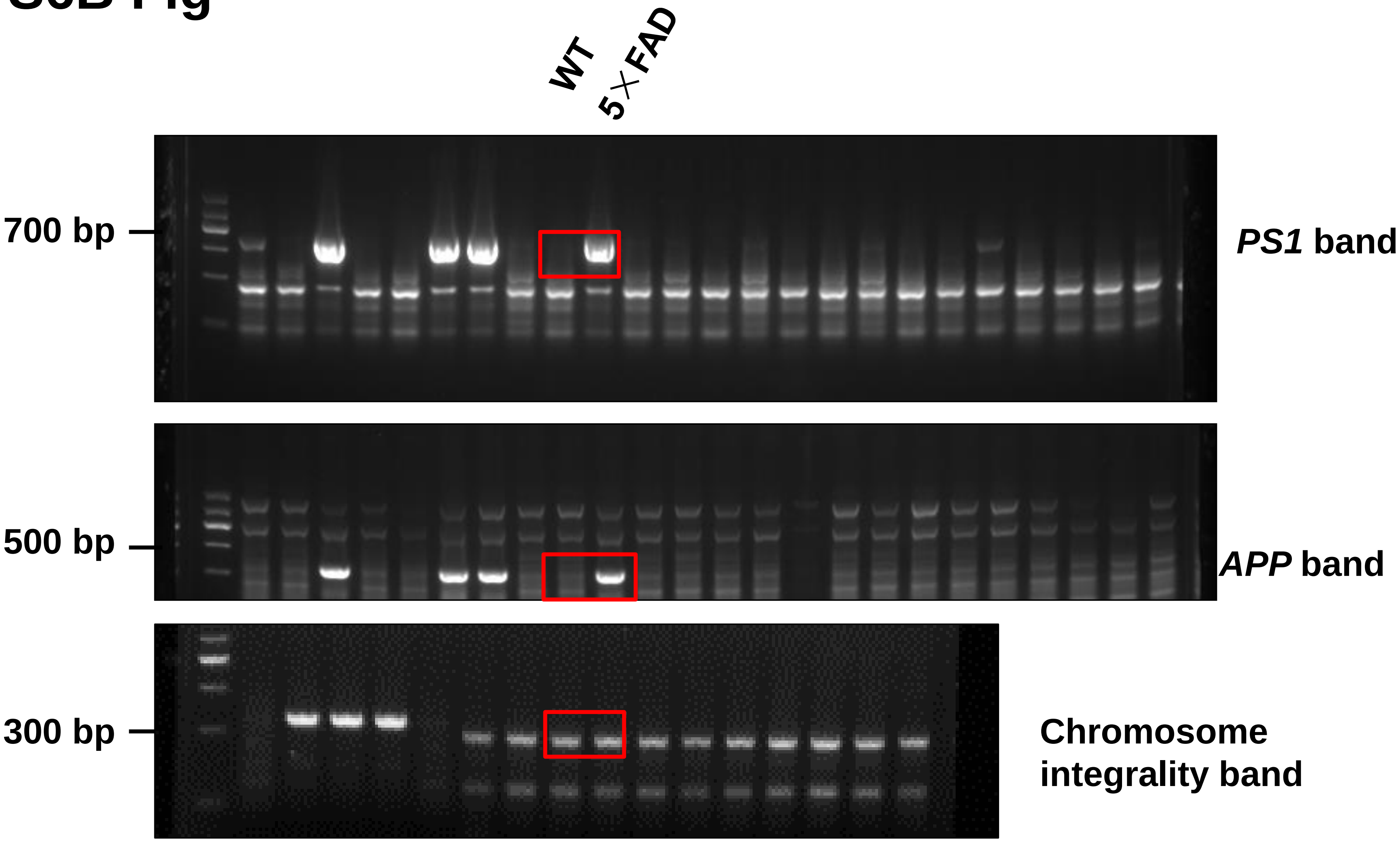

WT
5×FAD
700 bp
PS1 band
500 bp
APP band
300 bp
Chromosome integrality band

S6C Fig

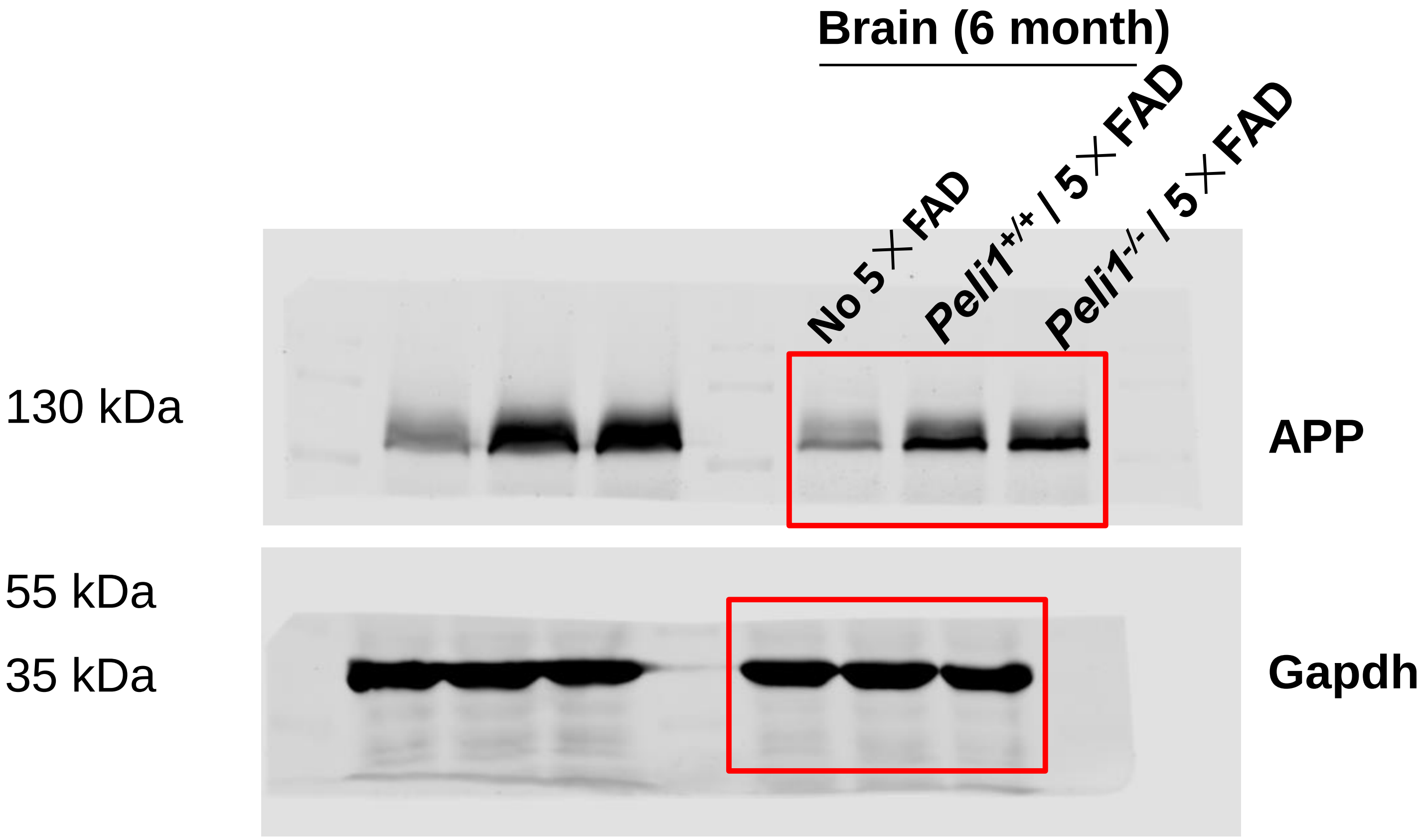
Brain (6 month)
No 5×FAD
Peli1+/+ / 5×FAD
Peli1-/- / 5×FAD
130 kDa
APP
55 kDa
35 kDa
Gapdh
